# Modular Design of Mitochondrion-Targeted Iron Chelators Allows Highly Selective Antiparasitic Activity against Trypanosomes and Apicomplexan Parasites

**DOI:** 10.1021/acsinfecdis.5c00548

**Published:** 2025-12-22

**Authors:** Ronald Malych, Yann Bordat, Kristýna Klanicová, Dominik Arbon, Farnaz Zahedifard, Anna Šipková, Eliška Drncová, Viktoriya Levytska, Jan Mach, Laura Plutowski-Wrobel, Marta Machado, Jan Štursa, Jaroslav Truksa, Markus Ganter, Daniel Sojka, Martin Zoltner, Sébastien Besteiro, Lukáš Werner, Robert Sutak

**Affiliations:** † Department of Parasitology, Faculty of Science, Charles University, BIOCEV, Vestec 25250, Czech Republic; ‡ 27037 LPHI, University of Montpellier, CNRS, INSERM, Montpellier 34095, France; § Department of Organic Chemistry, Faculty of Science, 37740 Charles University, Prague 25250, Czech Republic; ∥ Institute of Parasitology, 48311 Biology Centre of the Czech Academy of Sciences, BC CAS, Branišovská 1160/31, České Budějovice 37005, Czech Republic; ⊥ Centre for Infectious Diseases, Parasitology, 569852 Heidelberg University Hospital, Heidelberg 69120, Germany; # Graduate Program in Areas of Basic and Applied Biology, Instituto de Ciências Biomédicas Abel Salazar, 89239 Universidade do Porto, Porto 4050-313, Portugal; ∇ Laboratory of Clinical Pathophysiology, Diabetes Centre, Institute for Clinical and Experimental Medicine, Videnska 1958/9, Prague 140 21, Czech Republic; ○ Institute of Biotechnology of the Czech Academy of Sciences, BIOCEV, Vestec 25250, Czech Republic

**Keywords:** mitochondrion, iron chelators, antiparasitic agents, drug repurposing, *Trypanosoma*, *Toxoplasma*

## Abstract

Parasitic protozoa exhibit a high demand for iron, with mitochondrial iron metabolism representing a vulnerable target for chemotherapeutic intervention. We recently demonstrated that mitochondrial targeting of the iron chelator deferoxamine (DFO) via triphenylphosphonium (TPP) conjugation enhances its antiparasitic efficacy. To expand upon this strategy, mitochondrially targeted derivatives of DFO and deferasirox (DFX) were synthesized and evaluated for their activity against important human parasites. The DFX derivative mitoDFX was effective against *Trypanosoma* spp. and *Toxoplasma gondii* with remarkable selectivity. The fact that mitoDFX is a promising anticancer agent, which is likely safe to use in the context of human health, highlights the potential for drug repurposing in parasitology. Structure–activity relationship (SAR) studies and iron distribution analyses in trypanosomes revealed that mitochondrial targeting of the compounds, rather than iron chelation per se, is the main driver of the antiparasitic effects, underscoring the critical role of phosphonium salts in bioactivity.

## Graphical abstract

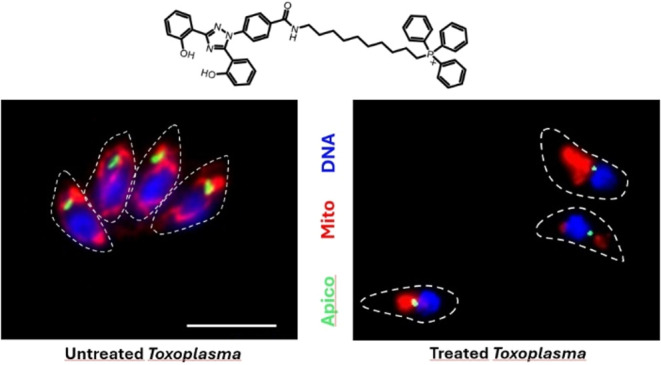

Despite major advances in the development of antiparasitic drugs, diseases caused by parasitic protozoa remain among the most significant threats to human and animal health. Therefore, the search for new therapeutic approaches must continue, especially in light of the emergence of resistance. One promising target is the mitochondrion, an essential organelle whose metabolic features and/or molecular machinery often vary significantly between the parasite and host, offering the potential for designing selective drugs.^1^ Mitochondria are very efficiently targeted by phosphonium salts, which accumulate in the organelle, attracted by a strong membrane potential.^2^ Derivatives of phosphonium salts have been shown to be highly effective, especially against kinetoplastids such as *Leishmania* and *Trypanosoma*, mosquito-transmitted *Plasmodium* species, and the distantly related apicomplexan order *Piroplasmida*, which includes the tick-borne pathogens *Babesia*.^3–8^ The importance of phosphonium salts in antiparasitic research has recently been highlighted in the case of mitochondrially targeted tamoxifen, an anticancer agent under clinical trial^9^ which has been proposed for drug repurposing due to its highly selective antiprotozoal activity.^8^ Diverse pharmacophores have been used in the design of new antiparasitic phosphonium salts, and in a previous study, we showed that the iron chelator deferoxamine (DFO), targeted to the mitochondria by two triphenylphosphonium (TPP) moieties linked by 10-carbon linker chains (mitoDFO), exhibits nanomolar potency against trypanosomes and *Plasmodium falciparum*. This is not unexpected, as mitochondria are at the core of iron metabolism and parasitic protists have a particularly high demand for this metal due to their rapid replication rates and the scarcity of bioavailable iron in the host environment. Indeed, iron chelators show antiparasitic effects at relevant concentrations and have long been considered potential antiparasitic therapeutics, especially in combination with other drugs, albeit their precise *in vivo* effects are not yet conclusive.^10^

In this context, we decided to broaden the spectrum of potential antiparasitic agents by introducing a new class of mitochondria-targeted chelators (mitochelators) consisting of a chelator, an alkyl linker, and a lipophilic cation derived from TPP. While DFO and deferasirox (DFX) are both approved for clinical use, DFO must be administered parenterally, whereas DFX is available as an oral formulation and may be more cost-effective, thus offering better prospects for future clinical use.^11^ Mitochondria-targeted DFX (mitoDFX, targeted to the organelle via a single TPP moiety) has recently been shown to have favorable anticancer effects.^12^ It inhibits tumor growth in both syngeneic and xenografted mouse models *in vivo*, while not significantly affecting systemic iron metabolism, hematological parameters, or body weight of the animals. It therefore provides a promising basis for repurposing as an antiparasitic drug.

Here, in addition to various DFO derivatives, we have synthesized and tested mitochondria-targeting derivatives of DFX and demonstrated their potential as highly potent and selective antiparasitic compounds against trypanosomes and apicomplexan parasites.

## Results

### Impact of MitoDFO Derivatives on Trypanosomes

Encouraged by the highly promising antiparasitic activity of the mitochondrially targeted iron chelator deferoxamine (mitoDFO) in our previous study, we synthesized 8 novel mitochondrially targeted DFO derivatives.^7^

The subject of modification was the mitochondrion-targeting TPP vector, while the iron-chelating moiety DFO was preserved. We reasoned that modulation of the vector properties might enhance the efficiency of transferring the DFO unit through the highly mitochondrial polarized membrane and modify the selectivity, given the differences in the molecular and physicochemical properties of host and parasite mitochondria. The approach primarily relied on the alteration of phosphonium cations in terms of their hydrophobicity and size. Additionally, modification of the length of the aliphatic linker was evaluated.

The results of testing their effect on *Trypanosoma brucei* are shown in Table 1 (compounds **1**–**10**). All derivatives showed some degree of selectivity toward trypanosomes. However, compared to mitoDFO (compound **2**), none of the modifications led to a significant increase in trypanocidal efficacy or an increase in selectivity. Nevertheless, these results offer insight into the influence of the mitochondrial anchor structure on the antiparasitic activity and selectivity of mitochelators. For instance, we showed that extending the linker by two carbons not only did substantially change the efficacy against trypanosomes but it also significantly reduced the selectivity (Table 1). Moreover, substitution of phenyl groups with alkyl groups led to a reduction in antiparasitic activity, with the most dramatic effect observed when even two of the three phenyl groups in the phosphonium moiety (compound **2**) were replaced with methyl groups (compound **9**) (∼20-fold decrease). Interestingly, the replacement of phosphorus with nitrogen in compound **3** resulted in a strong increase in activity compared with compound **4**, whereas the replacement of phenyl groups (compound **2**) with benzyl groups (compound **7**) resulted in a decrease in antiparasitic activity. We propose that the vector needs to be sufficiently hydrophobic to counterbalance the high hydrophilicity of DFO, thereby facilitating compound uptake and internalization through the inner membrane. The adverse impact of low lipophilicity is particularly evident with methyl substituents, which largely reduce the activity. The noticeable differences in activity and selectivity between tributylphosphonium and tributylammonium (**3** and **4**) suggest that while the ammonium vector is less efficient at crossing the inner membrane, it confers more favorable selectivity properties.

**Table 1:** Mean EC_50_ Values for DFO, DFX, and Their Mitochondrially Targeted Derivatives Derived from Dose–Response Curves for Human BJ Fibroblast and *T. brucei* (*n* ≥ 3, ±SE)

		BJ [nM]		*T. brucei* [nM]		
entry	compound	EC_50_	SE	EC_50_	SE	selectivity
1	triphenylphosphine-C10-DFO	>25,000	NA	881.5	17.7	NA
2	(triphenylphosphine-C10)2-DFO/**mitoDFO**	8910	3220	56.9	20.6	156
3	(tributylamine-C10)2-DFO	8140	3480	73.6	12.7	111
4	(tributylphosphine-C10)2-DFO	2840	710	281.4	91.8	10
5	(tricylohexylphosphine-C10)2-DFO	3080	290	52.0	17.9	59
6	(triphenylphosphine-C12)2-DFO	3630	1600	73.1	11.0	50
7	(tribenzylphosphine-C10)2-DFO	2550	830	185.8	45.2	14
8	(trioctylphosphine-C10)2-DFO	2920	310	133.9	24.8	22
9	(dimethylphenylphosphine-C10)2-DFO	>25,000	NA	1151.7	309.8	NA
10	(triphenylphosphine-C10-triazole)2-DFO	4130	450	133.9	24.8	31
11	triphenylphosphineBr-C10-norbenzoic-DFX	2110	570	5.5	0.2	384
12	triphenylphosphine-C8-DFX	1970	670	35.8	6.2	55
13	triphenylphosphine-C6-DFX	15,040	1520	49.8	19.7	302
14	triphenylphosphine-C10-DFX/**mitoDFX**	1710	530	4.2	0.2	412
15	triphenylphosphine-C10-NHCOBocFenylhydrazine	7400	2560	37.5	0.6	198
16	DFO	>25,000	NA	>25,000	NA	NA
17	DFX	>25,000	NA	8789.0	656.3	NA

### MitoDFX and Its Derivatives Are Efficient against Trypanosomes and Other Protozoan Parasites

Since the newly synthesized derivatives failed to significantly surpass the selectivity and potency of mitoDFO against *T. brucei*, we decided to focus on another chelator, DFX, whose mitochondrial derivative, mitoDFX, has recently been proposed as a promising new anticancer drug.^13^ In addition to mitoDFX, we synthesized two derivatives with a truncated alkyl linker (compounds **12** and **13**). We also designed one derivative in which we removed the carboxyphenyl group (compound **11**), as this is not required for iron chelation. In an attempt to understand the direct contribution of the iron-chelating effect to the antiparasitic activity of mitochondrially targeted DFX, we also tested a synthetic precursor devoid of chelating properties (compound **15**). As shown in Table 1, both mitoDFX (compound **14**) and compound **11** showed strong antiparasitic activity with EC_50_ values in the nanomolar range and high selectivity (>300) (Figure S2). Shortening the alkyl linker by two carbons significantly increased the EC_50_, and this trend continued slightly when eliminating further two carbons, but in this case, there was a large increase in selectivity (∼5.5-fold) due to a dramatic reduction in toxicity toward human cells. As expected, the loss of chelating ability led to a reduction in potency (EC_50_ = 37 nM for the nonchelating compound **15** versus 4 nM for mitoDFX), but the compound was still highly potent and selective (around 200-fold). It is therefore apparent that iron chelation is not the sole mechanism of action of these DFX derivatives.

Based on these results, we selected three compounds for further study: mitoDFX as the most selective mitochelator (compound **14**), compound **11** as a similarly selective and simplified mitochelator, as well as compound **15** as a nonchelating agent. We then tested these on a wider spectrum of pathogens, including further kinetoplastid and apicomplexan parasites. As can be seen from in Table 2, both mitoDFX and compound **11** are active against all microorganisms tested, achieving low-nanomolar EC_50_ values for African trypanosomes, *Leishmania mexicana*, and *Toxoplasma gondii*, with selectivity values in the hundreds. For *T. gondii*, which is an obligate intracellular parasite that develop within a parasitophorous vacuole, EC_50_ values were determined by monitoring the lytic cycle inside fibroblasts (Figure S1A,B). This also allowed us to visualize the impact of the compounds on the host cells in a higher concentration range (Figure S1A), in accordance with the EC_50_s determined for fibroblasts (Table 1). The lytic cycle of *T. gondii* involves successive rounds of host cell invasion, parasite replication, and egress,^14^ and compound effects during plaque assays are typically monitored over the course of a week. Thus, we next assessed the ability of the three compounds to rapidly and specifically impact the replication of *T. gondii* in its host cells and with a shorter treatment (24 h). All compounds led to a marked impact on parasite replication, with accumulation of vacuoles with fewer parasites (Figure S1C). This effect was particularly strong after treatment with mitoDFX, which thus seems to have the most immediate effect. Finally, for all parasites tested, note that compound **15**, which lacks the iron-chelating moiety, was still active, particularly against trypanosomes, although it was generally less effective than compound **11** and mitoDFX.

**Table 2:** Mean EC_50_ Values for Mitochondrially Targeted Derivatives of DFX (Compounds **11**, **14**, and **15**) Derived from Dose–Response Curves for the Parasites (*n* ≥ 3, ±SE)^a^

	compound **11**			compound **14** (mitoDFX)			compound **15**		
	EC_50_ [nM]	SE	selectivity	EC_50_ [nM]	SE	selectivity	EC_50_ [nM]	SE	selectivity
*T. brucei*	5.5	0.21	383	4.2	0.15	411	37.5	0.64	195
*T. gambiense*	7.5	0.35	282	10.4	<0.01	165	60.0	<0.01	122
*L. mexicana*	132.0	0.01	16	52.0	<0.01	33	348.0	0.01	21
*T. gondii*	5.0	<0.01	422	17.0	<0.01	101	350.0	0.05	21
*B. divergens*	17.2	1.10	122	41.2	1.17	42	76.2	1.19	96
*P. falciparum*	237.6	1.04	9	54.8	1.05	31	311.3	1.07	23

a Stages of parasites used: bloodstream forms of *Trypanosoma brucei* and *Trypanosoma gambiense*; axenic amastigotes of *Leishmania mexicana*; and intracellular stages of *Toxoplasma gondii*, *Babesia divergens* and *Plasmodium falciparum*.

### The Three Selected Compounds Impair Mitochondrial Function

To test whether the activity of these compounds is specifically directed toward mitochondrial function, we tested their effect on *T. brucei* as a representative of the Kinetoplastida and *T. gondii* as a representative of the Apicomplexa, as they are both relatively easy to propagate *in vitro*. In both cases, parasites were incubated for 24 h with compounds **11**, **15**, and mitoDFX at concentrations corresponding to three times the determined EC_50_. In *T. brucei*, mitochondrial membrane potential was measured using the cell-permeable red fluorescent dye TMRE.^8^ As shown in Figure 1A, all three compounds reduced the membrane potential of trypanosome mitochondria to less than 50%. To assess whether these compounds had an overall impact on *T. brucei* iron homeostasis, we monitored the distribution of iron incorporated into protein complexes in cells preincubated with the radioisotope ^55^Fe by native electrophoresis and phosphorimaging. As shown in Figure 1B, neither mitoDFX nor the nonchelating compound **15** did cause any detectable change in the overall protein-bound iron distribution upon 8 h exposure, unlike the nontargeted chelator DFO (Figure S3).

**Figure 1:**
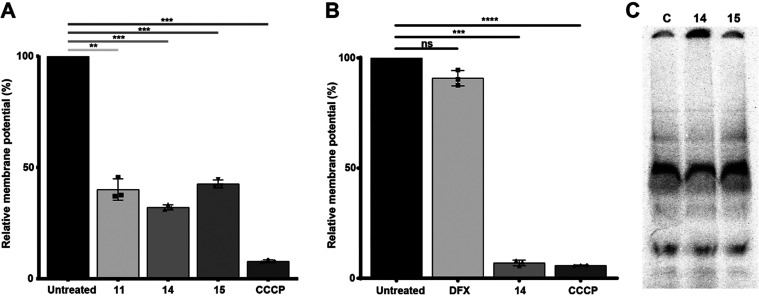
Effect of mitochondrially targeted DFX derivatives on *T. brucei* mitochondria and iron distribution. (A) Mitochondrial membrane potential of *T. brucei* after 24 h exposure to compound **11**, **14**, and **15** at concentrations equivalent to 3× EC_50_ and (B) comparison to the effect of DFX (16.5 nM—the same concentration as compound **14**). The CCCP (carbonyl cyanide *m*-chlorophenyl hydrazone) protonophore was used as a positive control. Values are mean ± standard deviation from *n* = 3 experiments, **p value ≤ 0.0; ***p value ≤ 0.001; and ****p value ≤ 0.0001 by one-way ANOVA. (C) *T. brucei* cells were grown in the presence of ^55^Fe-citrate as an iron source for 24 h (C) and, after washing, treated with compounds **14** and **15** for 8 h at concentrations corresponding to the EC_90_ values. Protein complexes were separated by blue native electrophoresis and ^55^Fe was visualized by phosphor imaging.

We next assessed the impact of the compounds on the mitochondrion of *T. gondii* after 24 h of treatment at 3 times the EC_50_. Using flow cytometric quantification of Mitotracker labeling, we found that all three compounds reduced the mitochondrial membrane potential, with **14** and **15** being the most impactful (Figure 2A). DFX used at the same concentration as its mitoDFX derivative (**14**) was found to have no specific impact. Microscopic observation confirmed the specificity of Mitotracker labeling (Figure 2B). As expected, treatment with the oxidative phosphorylation uncoupler FCCP (carbonyl cyanide *p*-trifluoromethoxyphenylhydrazone) led to a diffuse Mitotracker signal. Compound **11**, mitoDFX, and compound **15** led to a strong decrease in Mitotracker staining, although some residual signal was still found upon compound **11** treatment (Figure 2B, arrowheads). The mitochondrion is typically present as a single lasso-shaped organelle in *T. gondii*,^15^ but changes in mitochondrial morphology can be observed in response to drug treatment affecting the organelle.^15,16^ Strikingly, costaining between Mitotracker and a mitochondrial membrane marker showed that compounds **11**, **14**, and **15** altered the morphology of the organelle (Figure 2B). We quantified this more precisely by performing an immunofluorescence assay (IFA) after 24 h of treatment at 3 times the EC_50_ (Figure 2C,D). We confirmed that both mitoDFX and the simplified mitochelator caused the collapse of most *T. gondii* mitochondria, although the nonchelating derivative also had an effect. Interestingly, coimmunostaining of the apicoplast, another organelle of endosymbiotic origin hosted by the parasite, showed no detectable impact on this organelle under the same conditions (Figure 2C). Of note, treatment with DFX largely preserved both the morphology and the membrane potential of the mitochondrion. Overall, our results suggest that the three compounds specifically affect the mitochondrion in *T. gondii*.

**Figure 2:**
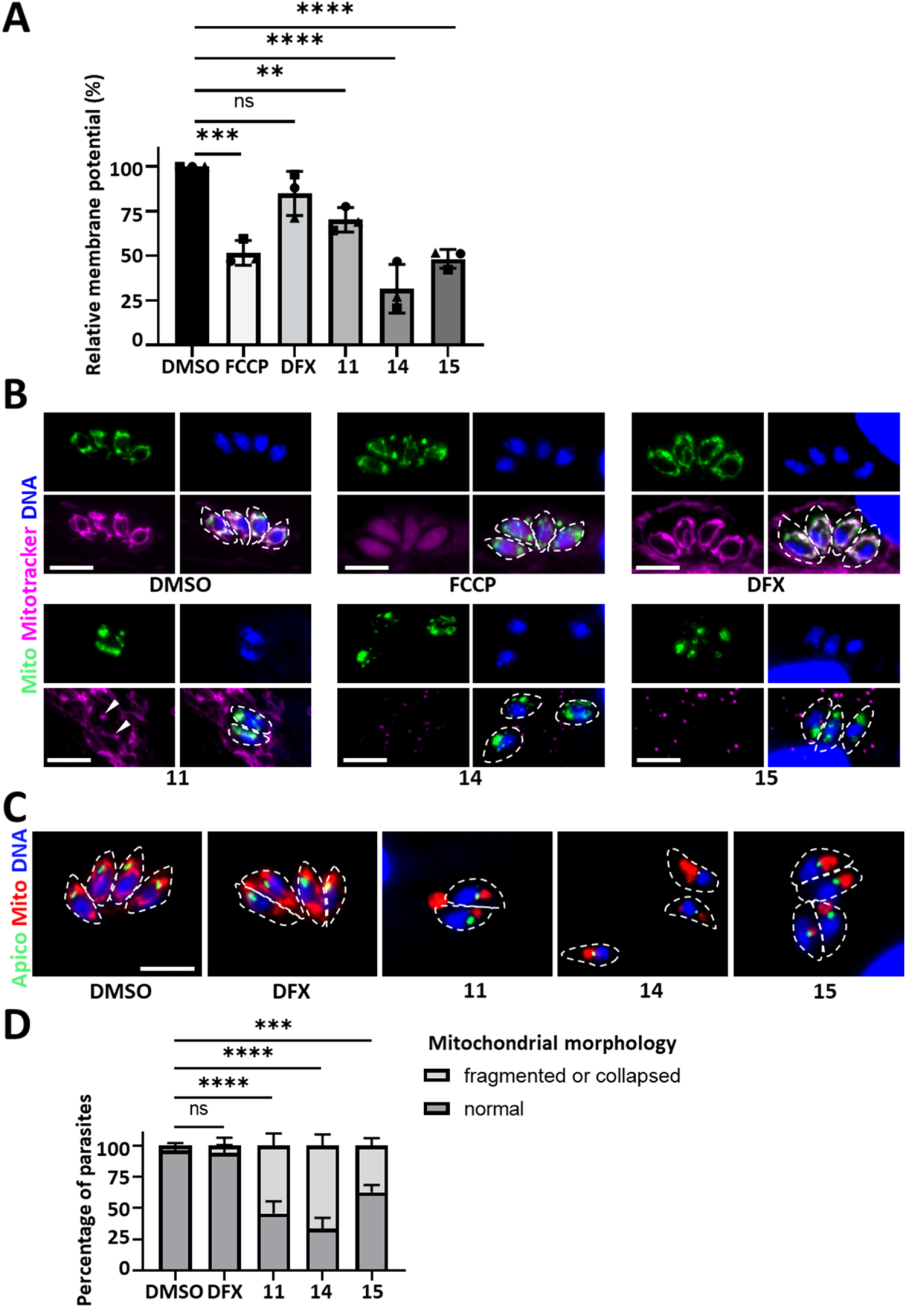
MitoDFX and simplified as well as nonchelating derivatives impact the *Toxoplasma* mitochondrion. (A) Parasites were treated with 3× EC_50_ of compounds **11**, **14**, and **15**, or with DFX (60 nM), or with DMSO vehicle control for 24 h and treated with mitochondrial potential-dependent fluorescent dye Mitotracker prior to analysis by cytometry. FCCP (carbonyl cyanide *p*-trifluoromethoxyphenylhydrazone), a potent uncoupler of oxidative phosphorylation, was used as a control. Data are mean ± standard deviation from *n* = 3 experiments. ns: not significant, **p value ≤ 0.01, ***p value ≤ 0.001, and ****p value ≤ 0.0001 by one-way ANOVA. (B) Parasites were treated as described in (A) and Mitotracker labeling (magenta) was visualized by fluorescence microscopy after costaining with a mitochondrial marker (green). Arrowheads show residual Mitotracker staining associated with mitochondria after treatment with compound **11**. DNA was stained with Hoechst (blue). Parasites are outlined with dashed lines. Scale bar represents 5 μm. (C) Parasites incubated with 3× EC_50_ of compounds **11**, **14**, and **15**, or with DFX (60 nM), or with DMSO vehicle control for 24 h and immunostained for the mitochondrion (red) and the apicoplast (green). DNA was stained with Hoechst (blue). Parasites are outlined with dashed lines. Scale bar represents 5 μm. (D) Quantification of the mitochondrial morphology change in parasites treated as described in (C), discriminating a normal lasso-shaped mitochondrial signal from a signal consistent with a fragmented/collapsed mitochondrion. Data are mean ± standard deviation from *n* = 3 experiments. ns: not significant, ***p value ≤ 0.001 and ****p value ≤ 0.0001 by one-way ANOVA.

## Discussion

This study aimed to investigate the antiparasitic potential of various mitochelators as a promising group of candidate agents. Modification of the previously described antiparasitic agent mitoDFO does not improve its selectivity but elucidates the structure–activity relationship (SAR) and thus provides insightful information on the mechanism of action of this family of compounds.

Regarding the mitoDFO SAR, we can draw a conclusion on two molecular properties affecting the potency and selectivity of the tested compounds. Our first observation is that the cytotoxicity toward mammalian cells (BJ) generally decreases with an increase of the compound’s overall hydrophobicity. Hydrophobicity can be predictably estimated based on the number of carbons in the substituent, with BJ EC_50_ values following the trend octyl > benzyl > cyclohexyl > phenyl > butyl. Substitution with methyl groups renders the compounds almost nontoxic or ineffective. The aliphatic linker also contributes to the overall hydrophobic interaction, thus rendering the compounds more toxic as the length of the aliphatic linker increases: C-6 < C-8 < C-10 < C-12. Therefore, we can conclude that the toxicity of the compounds is proportional to their hydrophobicity to some extent. However, hydrophobicity alone is not the primary determinant of activity against the model organism, as potency—and selectivity—varies significantly depending on substitution patterns. The most hydrophobic compounds exhibit notable selectivity, in the range of 20- to 50-fold compared to BJ EC_50_ values. Interestingly, less hydrophobic compounds containing triphenylphosphonium and tributylammonium moieties demonstrate both excellent selectivity and high potency. This observation highlights the importance of vector design in fine-tuning pharmacological properties.

Regarding DFX SAR, we can conclude that the cytoxicity of the compounds depends significantly on the length of the aliphatic linker making the compound with a C-6 linker by 1 order of magnitude less toxic toward BJ cells when compared to C-8 and C-10 derivatives. Nevertheless, all DFX derivatives have appreciable (20- to 50-fold) to excellent (up to 400-fold) selectivity, depending on the target parasite. The exact mechanism by which these compounds act on parasite mitochondria remains unclear, as it is likely multifactorial. It may not solely involve iron chelation. Iron is a central element in mitochondrial metabolism, being part of iron-based cofactors such as heme and Fe–S clusters, which are crucial for the optimal activity of mitochondrial electron transfer complexes. Iron chelation in the mitochondrion is thus likely to impact the respiratory capacity of the organelle. Fe–S proteins are, for instance, crucial for the mitochondrial electron transport chain of *T. gondii* and the fitness of the parasites.^17,18^ In trypanosomes, although mitochondria of bloodstream forms are metabolically suppressed, the parasite maintains a mitochondrial membrane potential^19^ which is, among other functions, important for the formation/export of Fe–S clusters for extra-mitochondrial proteins.^20^ This machinery for mitochondrial Fe–S export^21^ is thus essential for cytosolic or nuclear proteins, which are likely to be impacted by both chelation of mitochondrial iron and disruption of the mitochondrial membrane potential.

Previous studies have shown that phosphonium salts are highly effective even in the absence of the pharmacophore.^3,4^ The triphenylphosphonium moiety affects mitochondrial bioenergetics by inducing proton leak and uncoupling mitochondrial oxidative phosphorylation, thereby impacting ATP generation.^22^ Some chemical modifications to this moiety may allow targeting compounds to reach the mitochondrion without dissipating the mitochondrial membrane potential.^23^ However, in our case, it is difficult to measure the exact impact on mitochondrial metabolism solely due to iron chelation. Indeed, the nonchelating derivative of DFX showed generally lower antiparasitic activity but still displayed considerable efficacy, particularly against trypanosomes. Importantly, we failed to detect a noticeable impact on overall cellular iron homeostasis when monitoring iron incorporation into proteins after a short compound exposure of trypanosomes. However, we cannot completely exclude that Fe homeostasis is not impacted locally in the mitochondrion, and thus, this might also reflect a limited but specific impact on the organelle. Similarly, in *T. gondii*, while both the mitochondrion and the apicoplast harbor key iron-containing proteins in the form of cofactors, such as iron–sulfur (Fe–S) clusters,^18^ the morphological impact of the compounds is manifested primarily on the mitochondrion (Figure 2A,B). Again, if iron chelation mechanisms are at play, this suggests a local specific effect rather than an overall disruption of iron homeostasis. It is possible that mitochelators cause deficiency and/or mismetalation in some essential, yet low abundance, protein complexes in the mitochondrion.

*T. brucei* and *T. gondii* are two parasite models that are more amenable to phenotypic investigations, but our inhibition studies also highlighted a strong potential for the mitoDFX derivatives in other parasites responsible for a considerable economic and health burden. While *Plasmodium* species have long been in the spotlight due to their devastating impact on global human health, closely related apicomplexan parasites, such as *Babesia*, members of the piroplasmid group, also pose significant challenges. These parasites are responsible for economically important diseases in livestock, with *Babesia* infections alone contributing to substantial global economic losses, estimated in the billions of dollars annually.^24^ Due to their impact on both public and veterinary health, piroplasmids represent a critical target group for the development of novel antiparasitic therapies. Previous *in vitro* studies have demonstrated the inhibitory effects of iron chelators, such as CM1, against *Babesia bovis*, highlighting their potential as alternative treatments for bovine babesiosis.^25^ Interestingly, *N*-methylanthranilic desferrioxamine—a more lipophilic derivative of DFO, also known as a reversed siderophore—has been shown to permeate the plasma membrane of red blood cells and bind intracellular iron, leading to the inhibition of *P. falciparum* growth in culture.^26,27^ Our recent study shows a similar effect of the mitochondrially targeted DFO, rendering mitoDFO a potent and selective candidate drug for babesiosis therapy.^7^ One advantage of using organelle-targeted iron chelators over more general iron chelators is that they have the potential to enhance the impact on key local metabolic pathways and to improve specificity regarding host cells.

In conclusion, mitochondria-targeted chelators, particularly mitoDFO, mitoDFX, and TPPBr-C10-norbenzoic-DFX, exhibit potent and selective antiparasitic activity against Kinetoplastida and Apicomplexa. We demonstrate here that their action specifically impacts the mitochondrion, and while these chelators potentially act by disrupting the mitochondrial membrane potential, elucidating their mode of action calls for further studies. Our study demonstrates the great potential of modifying the phosphonium moiety and the alkyl linker. These modifications can dramatically change the effect of the chelator (or other fused pharmacophores) and achieve active concentrations substantially lower than those required for a biological effect in the parent pharmacophore. Importantly, both mitoDFO and mitoDFX are candidate anticancer agents with partially characterized pharmacological properties, which supports the promise of highly selective antiparasitic action described herein.^12,28^ Therefore, we propose mitochondrial iron chelators as promising leads for repurposing as antiparasitic drugs. Future studies will be conducted to optimize the mitochondrial anchor to improve the biodistribution and pharmacological properties.

## Methods

### Compound Synthesis

All synthesis procedures are given in Supporting File S4.

### Cell Cultures and Drug Sensitivity Assays

Human fibroblasts, *T. brucei*, *T. gambiense*, *P. falciparum*, *L. mexicana*, and *B. divergens* were cultivated as described previously, and dose–response curves were obtained by their cultivation on 96 well plates in a 2-fold seriel dilution of the appropriate drug.^7^

*T. gondii* tachyzoites of the RH strain were routinely maintained through passages in a human foreskin fibroblasts (HFFs) monolayer (ATCC CRL-1634). HFFs and parasites were cultured in standard Dulbecco’s Modified Eagle Medium (DMEM), supplemented with 5% decomplemented fetal bovine serum (FBS), 2 mM l-glutamine, 100 U/mL penicillin, and 100 μg/mL streptomycin (Gibco) at 37 °C and with 5% CO_2_ in an incubator with a controlled atmosphere.

The lytic cycle of *T. gondii* tachyzoites was assessed by a plaque assay as described previously.^29^ Briefly, tachyzoites were allowed to invade monolayers of HFFs for 3 h before addition of the compounds or the DMSO vehicle and were subsequently cultivated for 7 days at 37 °C and 5% CO_2_. They were then fixed with 4% (w/v) paraformaldehyde (PFA, diluted in phosphate-buffered saline (PBS)) for 20 min and stained with a 0.1% crystal violet solution (V5265, Sigma-Aldrich), washed, and air-dried before imaging under an Olympus MVX10 microscope.

### Mitochondrial Membrane Potential in *T. brucei*

The mitochondrial membrane potential of *T. brucei* 427 bloodstream form was assessed using the fluorescent probe TMRE (Thermo Fisher Scientific) as described in ref 7. Cells were preincubated for 24 h with 3× EC_50_ concentrations of compounds **11**, **14**, or **15**. Untreated cells and cells preincubated with a 50 μM CCCP uncoupler for 30 min were used as negative and positive controls, respectively. Approximately 1 × 10^6^ cells were incubated with 60 nM TMRE for 30 min, washed 1× with sterile PBS, and 5,000 events were measured on a Guava EasyCyte 8HT flow cytometer (Luminex) using a 488 nm excitation laser and a 583/26 nm detector. Median fluorescence was plotted against an untreated culture using Prism 8.0 (GraphPad Software), and statistical significance was determined using RM one-way ANOVA with Geisser–Greenhouse correction. The experiment was performed in three biologically independent replicates.

### Iron Chelation in *T. brucei*

The assay was performed as described in ref 30. *T. brucei* 427 bloodstream forms were grown in HMI-9 medium supplemented with 0.5 μM ^55^Fe (29 600 MBq mg^–1^) in the form of ferric citrate (1:20) and incubated for 24 h. After incubation with ^55^Fe, cells were washed with fresh HMI-9 medium and treated with compounds **14** and **15** for 8 h at concentrations corresponding to the EC_90_ values. Untreated cells were used as a control. After incubation, the cells were harvested by centrifugation and washed three times with NaCl–HEPES buffer (140 mM NaCl, 10 mM HEPES, pH 7.4). Cells were lysed by sonication in NaCl–HEPES buffer containing 1% digitonin and cOmplete EDTA-free protease inhibitor cocktail (Roche). Gels were vacuum-dried, autoradiographed for 5 days using a BAS-IP TR 2025 E tritium storage phosphor screen (GE Healthcare), and visualized with Typhoon FLA 7000 (GE Healthcare).

### *Toxoplasma* Replication Assay

Parasites were allowed to invade an HFF monolayer on coverslips for 3 h before addition of the compounds (at 3 times their respective EC_50_) and left to grow for 24 h before being fixed and performing parasites immunodetection was by IFA with a mouse anti-SAG1 antibody, as described previously.^31^ The number of parasites per vacuole was counted and scored. Independent experiments were conducted 3 times, and 200 random vacuoles were counted for each condition.

### *Toxoplasma* Mitochondrial Membrane Potential Measurements

We used the MitoTracker Deep Red FM fluorescent probe (Invitrogen) to assess the mitochondrial membrane potential^32^ in *Toxoplasma* tachyzoites. For each condition, 25 cm^2^ culture flasks containing HFFs were infected with approximately 4 × 10^6^ *T. gondii* tachyzoites, and 24 h later, compounds were added at 3 times their respective EC_50_ (for DFX, a concentration similar to the one used for mitoDFX, 60 mM, was used). After another 24 h of culture at 37 °C and 5% CO_2_, Mitotracker labeling was performed as described in the protocol provided by the manufacturer, with a 45 min incubation at 37 °C and 5% CO_2_ of the reconstituted dye diluted to 1/1000 in culture medium. Parasites were then fixed for 30 min in 4% (w/v) PFA (diluted in PBS), washed twice in PBS, scraped, syringed (with a 25 G needle), filtered on a 40 μm membrane, and resuspended in 1 mL of PBS. Parasites were then analyzed with an Aurora spectral cytometer (Cytek), using a 633 nm excitation laser and a 665 nm detector. 10,000 events were recorded. Mean fluorescence was plotted against vehicle-treated culture parasites using Prism 8.0 (GraphPad Software), and statistical significance was determined using one-way ANOVA with Tukey’s multiple-comparison test. The experiment was performed for three biologically independent replicates. For the membrane depolarization control, parasites were treated with 10 μM FCCP for 30 min prior to and during incubation with the Mitotracker dye.

For microscopic analysis of Mitotracker labeling, similar conditions were used, with the difference that coverslips (instead of flasks) were seeded with HFFs and were infected with *T. gondii* tachyzoites, and 3 h later, compounds were added (at 3 times their respective EC_50_). Intracellular parasites and the host cell monolayer were then fixed 24 h later with 4% (w/v) PFA (diluted in PBS) for 20 min, and after fixation, cells were permeabilized with 0.3% (v/v) Triton X-100 (diluted in PBS) for 10 min. Costaining with the anti-F1β ATPase mitochondrial marker was performed as described below.

### *Toxoplasma* Immunofluorescence Assay

For IFAs to assess organelle morphology, coverslips seeded with HFFs were infected with *T. gondii* tachyzoites, and 3 h later, compounds were added (at 3 times their respective EC_50_). Intracellular parasites and host cell monolayers were then fixed 24 h later with 4% (w/v) PFA (diluted in PBS) for 20 min. After fixation, cells were permeabilized with 0.3% (v/v) Triton X-100 (diluted in PBS) for 10 min. Coverslips were blocked with 2% (w/v) bovine serum albumin (BSA) for 1 h prior to immunolabeling with a primary antibody for 1 h. After 3 washes in PBS, a corresponding secondary antibody was incubated for 1 h. Coverslips were then incubated with 1 μg/mL of Hoechst stain for 5 min before 3 washes in PBS and, finally, mounted using Immu-Mount (Thermo Fisher) onto microscope slides. Primary antibodies used were prepared in 2% BSA (diluted in PBS) and used at the following concentrations: the apicoplast was stained using rabbit anti-PDH-E2 (1:500),^33^ the mitochondrion was stained using mouse anti-F1β ATPase (1:1000, gift of P. Bradley). Anti-mouse and anti-rabbit secondary antibodies were all from Thermo Fisher and were diluted at 1:4000. Image acquisition was performed with a Zeiss Axio Observer inverted microscope equipped with a Zeiss Axiocam 712 camera and 63×/1.4 or 100×/1.4 Oil Plan Achromat objectives. Images were processed with Zen Blue v3.6 software (Zeiss). *Z*-stack acquisitions were processed by a maximum-intensity orthogonal projection. Adjustments of brightness and contrast were applied uniformly, and paired images were acquired with the same exposure time. Quantifications of mitochondrial morphology were performed by microscopic observation of a series of at least 100 parasites, and data from three independent biological replicates was acquired and plotted using Prism 8.0 (GraphPad Software). Statistical significance was determined using one-way ANOVA with Tukey’s multiple-comparison test.

## Supplementary Material



## Notes

EC_50_ determination of selected compounds in *Toxoplasma* (Figure S1); EC_50_ curves for the tested compounds against selected parasitic protists (Figure S2); effect of nontargeted chelator on iron-containing proteins of *Trypanosoma* (Figure S3); synthesis methods of tested compounds (File S4) (PDF)
