# Supplementary material for: Modular Design of Mitochondrion-Targeted Iron Chelators Allows Highly Selective Antiparasitic Activity against Trypanosomes and Apicomplexan Parasites
Source: ACS Infect Dis. 2025 Dec 22;12(1):119–27. doi: 10.1021/acsinfecdis.5c00548 (PMC12797238; doi:10.1021/acsinfecdis.5c00548)
Supplement: Supplementary file 1 [file id5c00548_si_001.pdf]

Supporting Information

Title: Modular design of mitochondrion-targeted iron chelators allows highly selective antiparasitic activity against trypanosomes and apicomplexan parasites

List of authors:

Ronald Malych - Department of Parasitology, Faculty of Science, Charles University, BIOCEV, Vestec, 25250, Czech Republic

Yann Bordat - LPHI, University of Montpellier, CNRS, INSERM, Montpellier, France

Kristýna Klanicová - Department of organic chemistry, Faculty of Science, Charles University, Prague, Czech Republic

Dominik Arbon - Department of Parasitology, Faculty of Science, Charles University, BIOCEV, Vestec, 25250, Czech Republic

Farnaz Zahedifard - Department of Parasitology, Faculty of Science, Charles University, BIOCEV, Vestec, 25250, Czech Republic

Anna Šipková - Department of Parasitology, Faculty of Science, Charles University, BIOCEV, Vestec, 25250, Czech Republic

Eliška Drncová - Department of Parasitology, Faculty of Science, Charles University, BIOCEV, Vestec, 25250, Czech Republic

Viktoriya Levytska - Institute of Parasitology, Biology Centre of the Czech Academy of Sciences, BC CAS, Branišovská 1160/31, České Budějovice 37005, Czech Republic.

Jan Mach - Department of Parasitology, Faculty of Science, Charles University, BIOCEV, Vestec, 25250, Czech Republic

Laura Plutowski-Wrobel - Centre for Infectious Diseases, Parasitology, Heidelberg University Hospital, Heidelberg, 69120, Germany

Marta Machado - Centre for Infectious Diseases, Parasitology, Heidelberg University Hospital, Heidelberg, 69120, Germany
- Graduate Program in Areas of Basic and Applied Biology, Instituto de Ciências Biomédicas Abel Salazar, Universidade do Porto, Porto 4050-313, Portugal

Jan Štursa - Laboratory of Clinical Pathophysiology, Diabetes Centre, Institute for Clinical and Experimental Medicine, Videnska 1958/9, 140 21 Prague, Czech Republic.

Jaroslav Truksa - Institute of Biotechnology, Czech Academy of Sciences, BIOCEV, Vestec, 25250, Czech Republic

Markus Gantner - Centre for Infectious Diseases, Parasitology, Heidelberg University Hospital, Heidelberg, 69120, Germany

Daniel Sojka - Institute of Parasitology, Biology Centre, Academy of Sciences of the Czech Republic,

Branišovská 1160/31, České Budějovice, 37005, Czech Republic

Martin Zoltner - Department of Parasitology, Faculty of Science, Charles University, BIOCEV, Vestec, 25250, Czech Republic

*Sébastien Besteiro - LPHI, University of Montpellier, CNRS, INSERM, Montpellier, France

*Lukáš Werner - Laboratory of Clinical Pathophysiology, Diabetes Centre, Institute for Clinical and Experimental Medicine, Videnska 1958/9, 140 21, Prague, Czech Republic

*Robert Sutak - Department of Parasitology, Faculty of Science, Charles University, BIOCEV, Vestec, 25250, Czech Republic

*Corresponding authors: sutak@natur.cuni.cz, sebastien.besteiro@umontpellier.fr, wernerlukas@yahoo.com

Figure S1

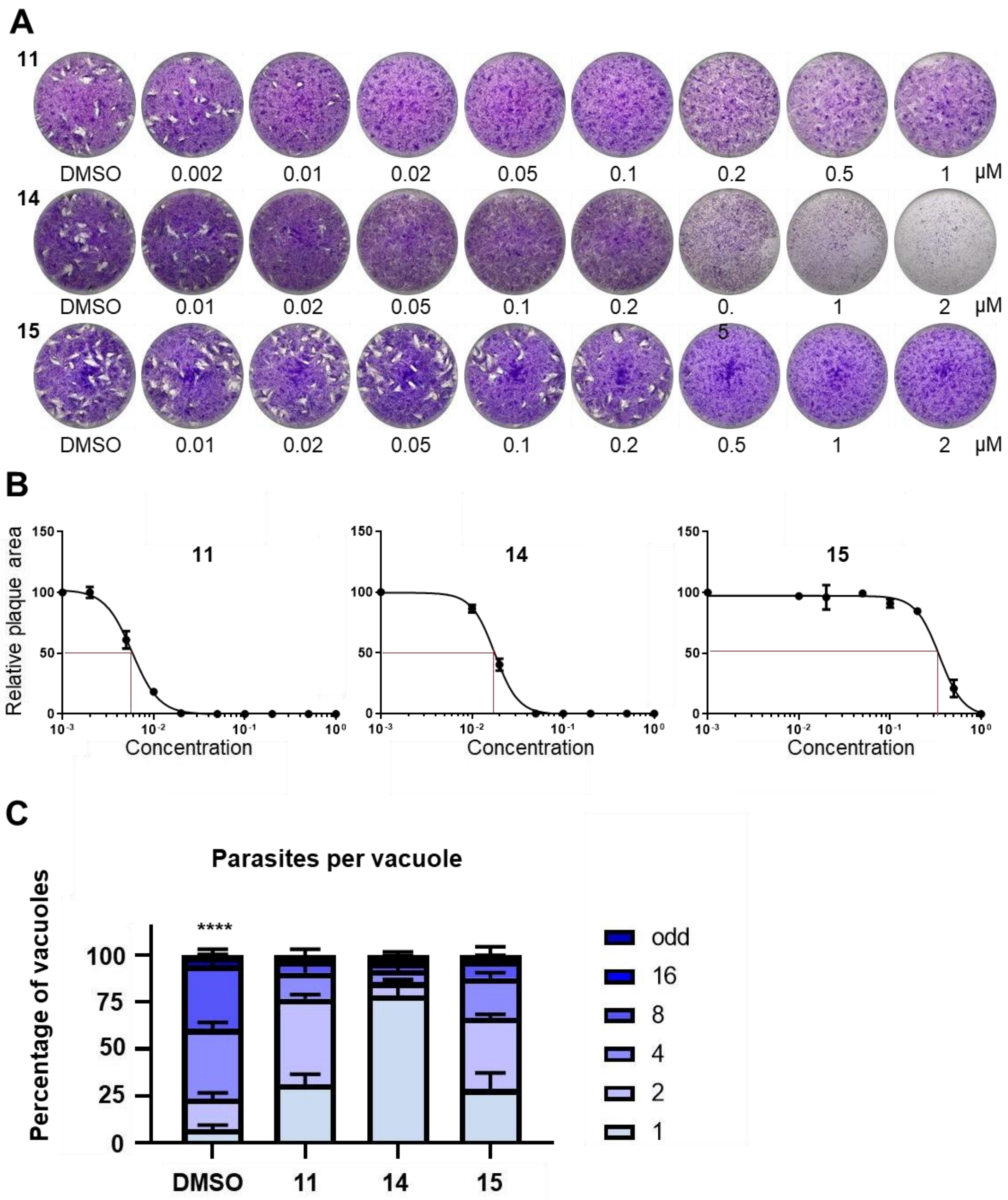

Figure S1. EC50 determination of selected compounds in *Toxoplasma*.

A) Representative plaque assays for evaluating the impact of the compounds on the lytic cycle of *T. gondii*. Plaque assays were carried out by infecting HFF monolayers with *T. gondii* tachyzoites and leaving them to develop for 7 days in the presence of increasing concentration of compounds. The DMSO vehicle-treated parasites were used as a control. **B**) EC50 curves based on plaque area measured from three independent plaque assays. Data are mean ± standard deviation. **C**) Replication assay of *T. gondii* tachyzoites, which were allowed to invade HFF-coated coverslips, then treated for 24 h with the compounds at 3 times the EC50 or DMSO vehicle, after which number of parasites per vacuole was quantified for each condition and expressed as a percentage, 200 vacuoles were counted for each condition. Values represented are mean ± standard deviation of *n*=3 independent biological replicates, **** p value ≤ 0.0001 showing statistically significant difference for the 1, 2, 4, and 8 parasites per vacuole categories between the DMSO control and the compounds, using two-way ANOVA with Dunnett's multiple comparison test.

Figure S2

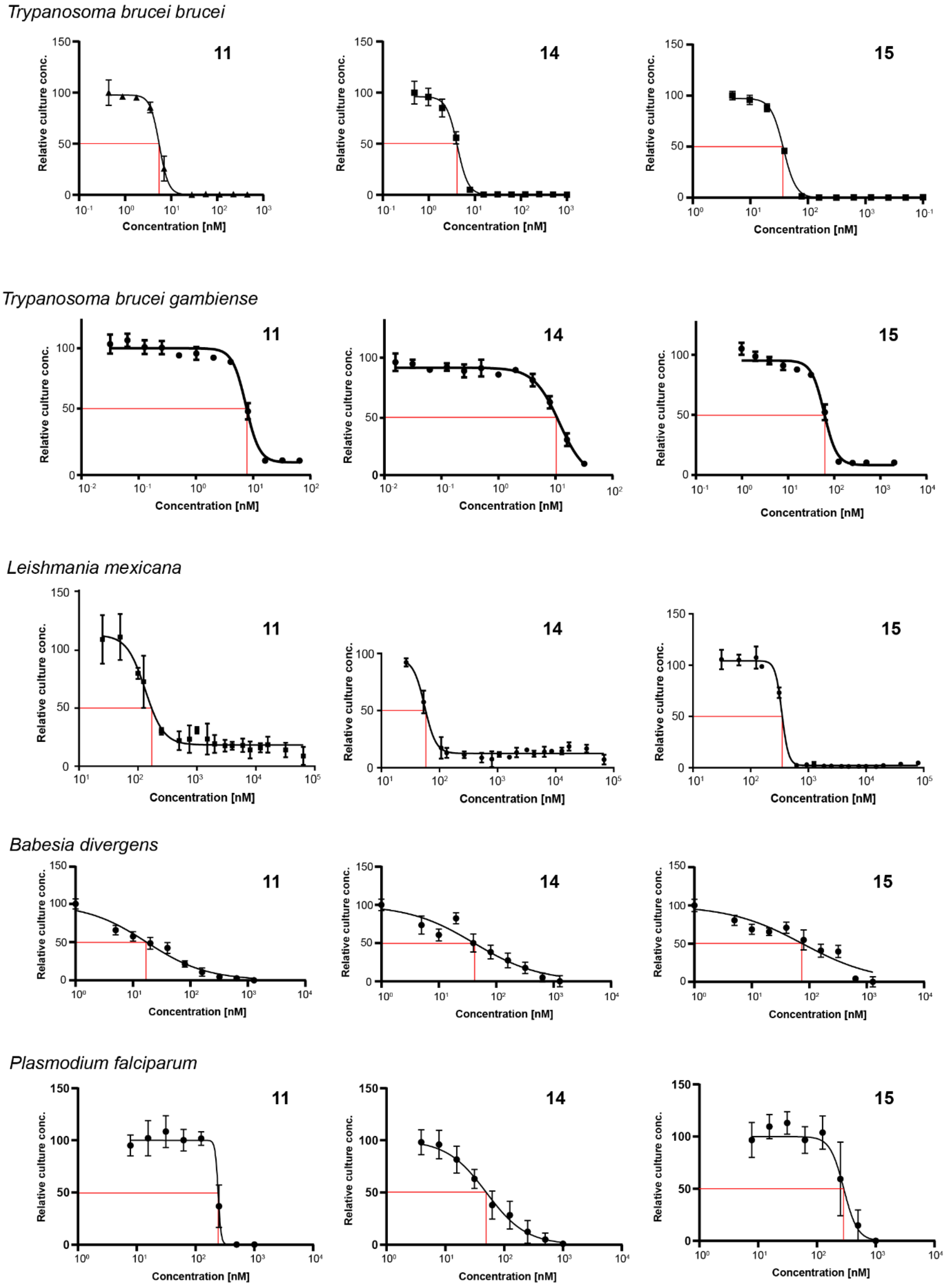

Figure S2. EC_{50} determination of selected compounds in parasitic protists. EC_{50} curves measured from three independent drug sensitivity assays. Data are mean ± standard deviation.

Figure S3

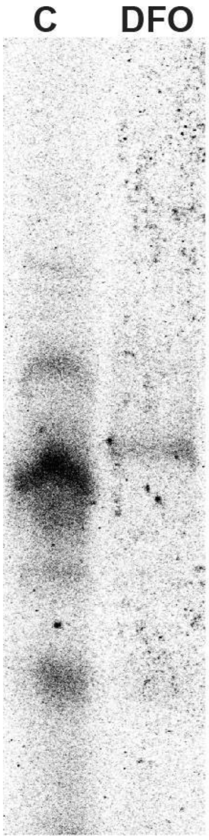

Figure S3. Effect of non-targeted chelator on iron containing proteins of *Trypanosoma*. *T. brucei* cells were grown in the presence of ^{55}Fe-citrate as an iron source for 24 h (C) and, after washing, treated with 20 μM deferoxamine (DFO) for 8 h. Protein complexes were separated by blue native electrophoresis and ^{55}Fe was visualised by phosphorimaging.

Supplementary File S4

Compounds synthesis methods

Compound 1

triphenyl(3,14,25-trihydroxy-2,10,13,21,24-pentaoxo-3,9,14,20,25,31-hexaazahentetracontan41-yl)phosphonium

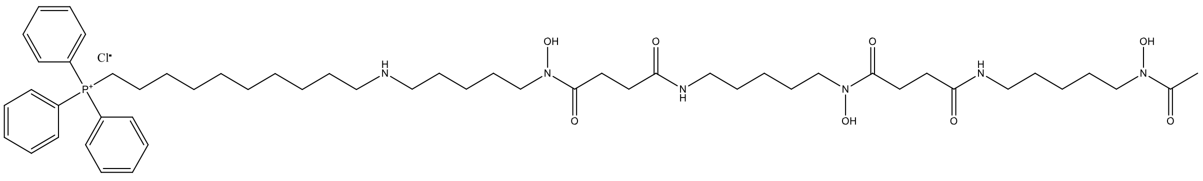

DMF (1 mL) was added to a flask charged with deferoxamine mesylate (50 mg, 0.076 mmol), (10bromodecyl)triphenylphosphonium bromide (100 mg, 0.178 mmol) and sodium bicarbonate (64 mg, 0.762 mmol). Reaction mixture was stirred and heated to 60°C after 4 hours was the heater turned off and stirring continued 18 hours at room temperature. The mixture was diluted with dichloromethane (10 mL), filtered and concentrated under vacuum. The resulting oil was triturated with diethylether (10 mL) and precipitate collected. The precipitate was than dissolved in methanol (3 mL), filtered through ion-exchange resin (3.6g, Dowex 2 x 10-Cl^-) and concentrated under vacuum. The crude product was submitted to chromatography (10 mL of Silica, chloroform/methanol/ammonia 100:5:2→100:10:2→100:15:2) to give 15 mg of slightly yellow product.

R_f 0.07 ($CHCl_3$/CH_3OH/NH_3 100:10:2);
^{1}H NMR (500 MHz, CD_3OD) δ 7.93 – 7.87 (m, 3H), 7.85 – 7.72 (m, 12H), 3.64 – 3.56 (m, 6H), 3.45 – 3.36 (m, 2H), 3.17 (t, *J* = 6.6 Hz, 4H), 2.77 (t, *J* = 7.0 Hz, 4H), 2.60 (dd, *J* = 15.4, 9.2 Hz, 2H), 2.51 – 2.38 (m, 6H), 2.10 (s, 3H), 1.74 – 1.59 (m, 8H), 1.59 – 1.40 (m, 10H), 1.40 – 1.19 (m, 16H).
^{13}C NMR (126 MHz, cd_3od) δ 174.90, 174.89, 174.07, 173.99, 172.98, 136.27 (d, *J* = 3.0 Hz), 134.79 (d, *J* = 10.0 Hz), 131.51 (d, *J* = 12.5 Hz), 120.00 (d, *J* = 86.3 Hz), 55.12, 54.70, 50.52, 50.31, 49.84, 40.27 (2C), area of overlaping signals, some of them have *J* coupling with phosphorus - 31.60, 31.55, 31.53, 30.62, 30.54, 30.52, 30.43, 30.37, 30.32, 30.05, 29.98, 29.94, 29.89, 29.85, 29.62, 28.95, 28.91, 28.71, 28.33, 27.43, 27.37, 25.28, 24.95, 24.91, 23.54 (d, *J* = 4.4 Hz), 22.88, 22.47, 20.21.
IR-3400,3303, 3093, 3054, 2927, 2854, 1642, 1622, 1588, 1566, 1481, 1461, 1438, 1373, 1252, 1162, 1113, 996, 746, 723, 691é
HR-MS: m/z = 1, found: 961.59155, calcd. for $C_{53}H_{82}N_6O_8P^+$: 961.59263
HR-MS: m/z = 2, found: 481.29965, calcd. for $C_{53}H_{83}N_6O_8P^{2+}$: 481.29632

Compound 2

triphenyl(3,14,25-trihydroxy-2,10,13,21,24-pentaoxo-31-(10-(triphenylphosphonio)decyl)
3,9,14,20,25,31-hexaazahentetracontan-41-yl)phosphonium

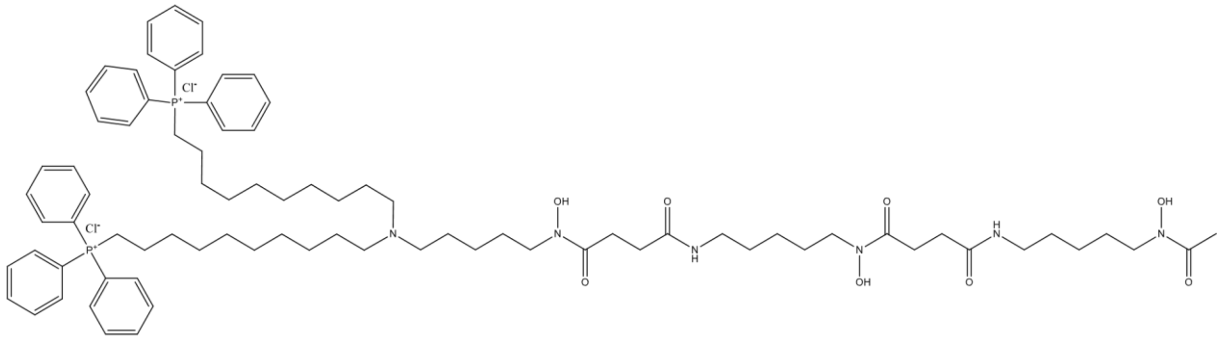

Chemical Formula: $C_{81}H_{116}Cl_2N_6O_8P_2$
Molecular Weight: 1434,67666

DMF (2 mL) was added to a flask charged with deferoxamine mesylate (100 mg, 0.152 mmol), (10-bromodecyl)triphenylphosphonium bromide (200 mg, 0.356 mmol) and sodium bicarbonate (600 mg, 7.143 mmol). Reaction mixture was stirred and heated to 70°C after 4 hours was the heater turned off and stirring continued 18 hours at room temperature. The mixture was diluted with dichloromethane (20 mL), filtered and concentrated under vacuum. The resulting oil was triturated with diethylether (12 mL) and resulting precipitate again triturated with petrolether (12 mL). Residue was than dissolved in methanol (3 mL), filtered through ion-exchange reisin (7 g, Dowex 2 x 10-Cl^-) and concentrated under vacuum. The crude product was submitted to chromatography (10 mL of Silica, chloroform/methanol/ammonia 100:10:1(200 mL)→100:15:1.5(200 mL)) to give 48 mg of slightly yellow product.

R_f 0.05 ($CHCl_3$/CH_3OH/NH_3 100:10:2);
^{1}H NMR (500 MHz, CD_3OD) δ 7.93 – 7.86 (m, 6H), 7.86 – 7.71 (m, 24H), 3.63 – 3.54 (m, 6H), 3.47 – 3.37 (m, 4H), 3.20 – 3.12 (m, 4H), 2.77 (t, *J* = 6.3 Hz, 4H), 2.55 (dd, *J* = 15.9, 8.9 Hz, 4H), 2.46 (t, *J* = 6.8 Hz, 4H), 2.09 (s, 3H), 1.74 – 1.60 (m, 12H), 1.60 – 1.44 (m, 14H), 1.42 – 1.19 (m, 26H).
^{13}C NMR (126 MHz, cd_3od) δ 174.8, 174.4, 174.3, 173.3, 136.24 (d, *J* = 3.0 Hz), 134.78 (d, *J* = 10.0 Hz), 131.50 (d, *J* = 12.6 Hz), 119.98 (d, *J* = 86.3 Hz), 55.0, 54.6, 40.2, 31.7-29.8 area of overlaping signals, 31.07 – 29.51 (m), 28.9, 28.5, 27.3, 26.9, 25.5, 24.9 (2C), 23.5(2C), 22.9, 22.4, 20.2.
IR-3264, 3059, 1636, 1588, 1547, 1485, 1457, 1439, 1362, 1259, 1114, 996, 750, 729, 691
HR-MS: m/z = 2, found: 681.41589, calcd. for $C_{81}H_{116}N_6O_8P^{2+}$: 681.415945
HR-MS: m/z = 3, found: 454.61316, calcd. for $C_{81}H_{117}N_6O_8P^{3+}$: 454.613056

<u>Compound 3</u>

N1,N1,N1-tributyl-N10-(10-(tributylammonio)decyl)-N10-(3,14,25-trihydroxy-2,10,13,21,24pentaoxo-3,9,14,20,25-pentaazatriacontan-30-yl)decane-1,10-diaminium trichloride

Tri(n-butyl)bromodecylamonium bromide (1,41g mg; 2,9 mmol; 10 eq.), deferoxamine mesylate salt (190 mg; 0,286 mmol; 1eq.) and $NaHCO_3$ (1,15 g; 0,014 mol; 47 eq.) were dissolved in dry DMF (20 ml) and heated to 60°C while stirred for 4h. After that reaction was cooled to rt and stirred overnight. Reaction progress was monitored by TLC ($CHCl_3$/MeOH/NH_3; 80/20/2). Reaction was diluted with 20 ml of DCM, $NaHCO_3$ was filtered off and solvents were evaporated. Crude product was diluted in dichloromethane (5 mL) and precipitated by addition into ice cooled Et_2O (40 ml) and PE (40 ml). After two hours of stirring was solvent decanted off and precipitate was dissolved in methanol/H_2O (5 ml) and filtrated through DOWEX (45 ml). Solvents were evaporated and product was purified by column chromatography on silica gel
($CHCl_3$/MeOH/NH_3 100/10/1). Reaction afforded yellow oil of the formula 7 (24 mg, 25 %).

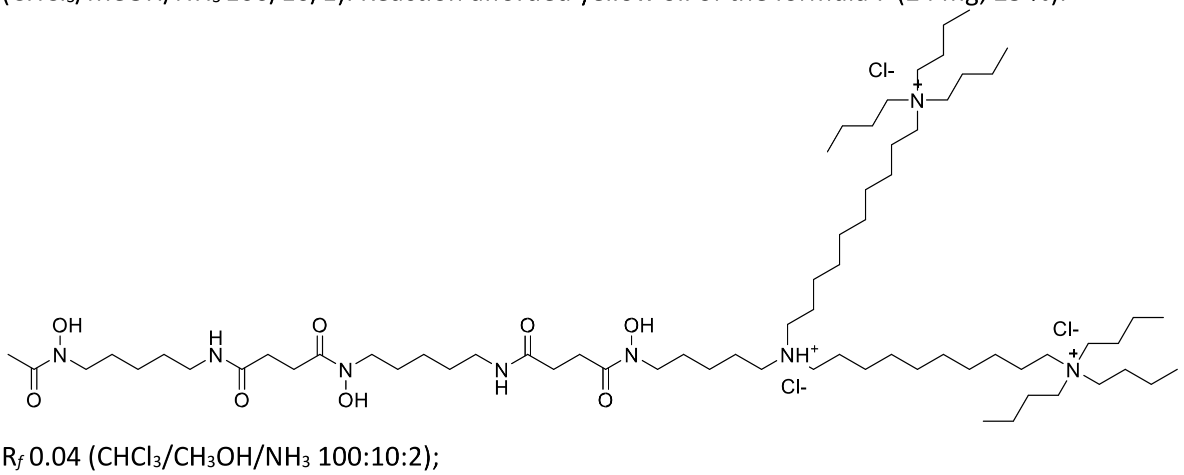

R_f 0.04 ($CHCl_3$/CH_3OH/NH_3 100:10:2);
^{1}H NMR (500 MHz, Methanol-d4) δ 3.49 (t, J = 7.1 Hz, 6H), 3.18 – 3.10 (m, 16H), 3.10 – 3.04 (m, 6H), 3.01 (t, J = 8.3 Hz, 4H), 2.66 (t, J = 7.2 Hz, 4H), 2.36 (q, J = 7.4 Hz, 4H), 1.99 (s, 3H), 1.65 – 1.48 (m, 26H), 1.47 – 1.36 (m, 8H), 1.39 – 1.16 (m, 40H), 0.92 (t, J = 7.4 Hz, 18H).
^{13}C NMR (126 MHz, Methanol-d4) δ 174.46 (overlap) , 170.94 (overlap), 59.71 , 59,68 , 59,67 , 59,52 , 59,50 , 40.29 , 31,44 , 30,93 , 30,76 , 30,65 , 30,55 , 30,48 , 30,24 , 30,03 , 28.93 , 27,42 , 27.35 , 24,90 , 24.81 , 22.81 , 20.73 , 18.38 , 18,23 , 18,08 , 13.97 .
173.4, 173.0, 58.27(overlap), 58.07(overlap), 38.85, 30.07, 29.19, 29.12, 29.08, 28.78, 28.56,

27.51, 25.98, 25.92, 23.49, 23.39, 21.38, 19.30., 12.55
MS: Calculated: 604,53916 Found: 604,53898 (M/Z=2)
IR: 3428, 3079, 1636, 1546, 1114, 879

Compound 4

42,42-dibutyl-3,14,25-trihydroxy-2,10,13,21,24-pentaoxo-31-(10-(tributylphosphonio)decyl)-3,9,14,20,25,31-hexaaza-42-phosphahexatetracontane-31,42-diium trichloride

Tri(n-butyl)bromodecyl phosphonium bromide (1,45g mg; 2,9 mmol; 10 eq.), deferoxamine mesylate salt (190 mg; 0,286 mmol; 1eq.) and $NaHCO_3$ (1,15 g; 0,014 mol; 47 eq.) were dissolved in dry DMF (20 ml) and heated to 60°while stirred for 4h. After that reaction was cooled to rt and stirred overnight. Reaction progress was monitored by TLC ($CHCl_3$/MeOH/NH_3; 80/20/2). Reaction was diluted with 20 ml of DCM, $NaHCO_3$ was filtrated off and solvents were evaporated. Crude product was dissolved in dichloromethane (5 mL) and precipitated by addition into ice cooled Et_2O (40 ml) and PE (40 ml). After two hours of vigorous stirring was solvent decanted off and precipitate was dissolved in methanol/H_2O (5 ml) and filtered through DOWEX (45 ml). Solvents were evaporated. Product was purified by column chromatography on silica gel ($CHCl_3$/MeOH/NH_3 100/10/1). Reaction afforded yellow oil of the formula 6 (22 mg, 22 %).

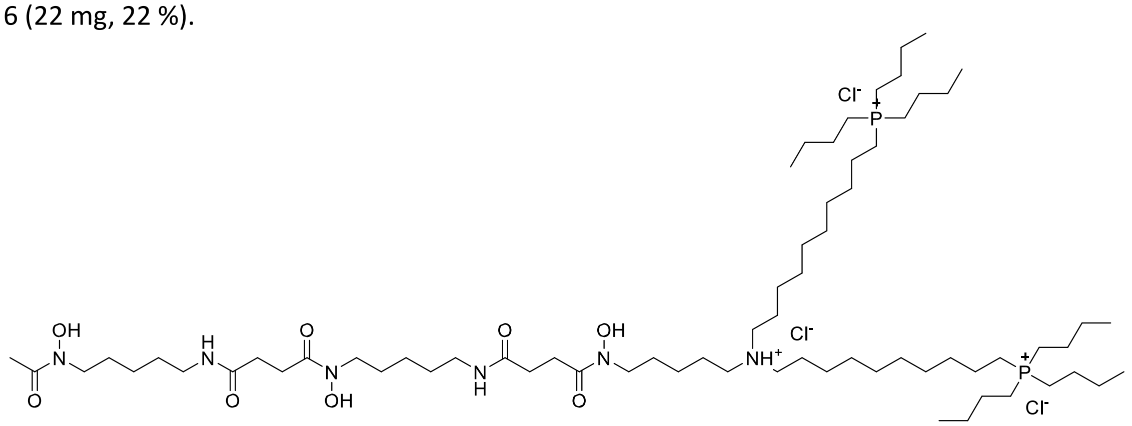

R_f 0.04 ($CHCl_3$/CH_3OH/NH_3 100:10:2);
^{1}H NMR (500 MHz, Methanol-d4) δ 3.70 – 3.56 (m, 6H), 3.24 – 3.13 (t, J = 7.2 Hz ,4H), 2.83 (s, 4H), 2.78 (t, J = 7.2 Hz, 4H), 2.47 (q, J = 7.0 Hz, 4H), 2.31 – 2.14 (m, 16H), 2.11 (s, 3H), 1.71-1.59 (m, 16H), 1.59 – 1.44 (m, 30 H), 1.44-1.27 (m, 28H), 1.02 (t, J = 7.0 Hz, 18H).
^{13}C NMR (126 MHz, Methanol-d_4) δ 173.41, 173.09 , 173.03 , 172.04 , 53.29 , 52.91 , 38.86 , 30.47 (d, *J* = 15.2 Hz), 30.07 , 30.01 , 29.21 , 29.06 , 28.59 , 28.57 , 27.52 , 27.45 , 26.81 , 25.93 , 23.55 (d, *J* = 15.4 Hz), 22.96 (d, *J* = 4.3 Hz), 20.99 , 20.96 , 17.95 (d, J = 48.0 Hz) , 17.65 (d, *J* = 47.8 Hz), 12.28
MS: Calculated: 621,50985 Found: 621,51044 (M/Z=2)
IR: 3066, 2930, 2857, 1640, 1550, 1101, 720

Compound 5

Tricyclohexyl(3,14,25-trihydroxy-2,10,13,21,24-pentaoxo-31-(10-(tricyclohexylphosphonio) decyl)-3,9, 14,20,25,31-hexaazahentetracontan-31-ium-41-yl)phosphonium trichloride

Bromodecyltricyclohexylphosphonium bromide (1,66g mg; 2,9 mmol; 10 eq.), deferoxamine mesylate salt (190 mg; 0,286 mmol; 1eq.) and $NaHCO_3$ (1,13 g; 0,013 mol; 47 eq.) were dissolved in dry DMF and heated to 60°C while stirred for 4h. After that reaction was cooled to rt and stirred overnight. Reaction progress was monitored by TLC ($CHCl_3$/MeOH/NH_3; 80/20/2). When finished, reaction was diluted by 20 ml of DCM, $NaHCO_3$ was filtered off and solvents were evaporated. Crude product was dissolved in dichloromethane (5 mL) and precipitated by addition into in ice cooled Et_2O (40 ml) and PE (40 ml). After 1-2 hrs of vigorous stirring was solvent decanted off and resulting precipitate was dissolved in methanol/H_2O (5 ml) and filtrated through DOWEX (45 ml). Solvents were evaporated and product was purified by column chromatography on silica gel ($CHCl_3$/MeOH/NH_3 100/10/1). Reaction afforded yellow oil of bisphosphonium deferoxamine of the formula 4 (148 mg, 35 %) and monophosphonium deferoxamine of the formula 5 (59 mg, 48 %).

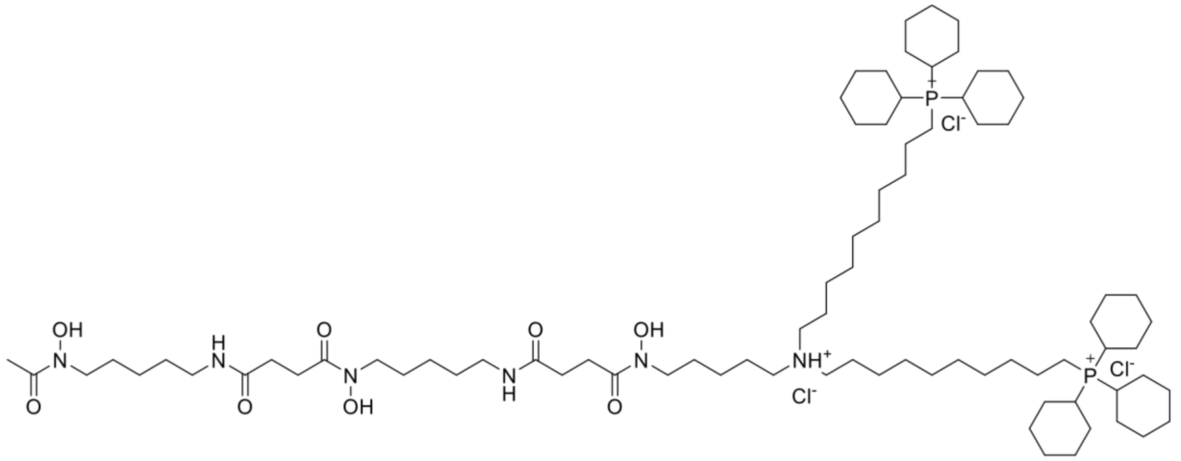

R_f

0.08 ($CHCl_3$/CH_3OH/NH_3 100:10:2)

^{1}H NMR (500 MHz, Methanol-d4) δ, 3.68 – 3.58 (m, 6H), 3.23 – 3.12 (t, J = 6.8 Hz 4H), 2.78 (t, J = 7.2 Hz, 6H), 2.54(m, 6H), 2.48 (m, 4H) , 2.31 – 2.20 (m, 4H), 2.11 (s, 3H), 2.07 – 1.88 (m, 24H), 1.87 – 1.76 (m, 6H), 1.72 – 1.33 (m, 84H).

^{13}C NMR (126 MHz, Methanol-d4) δ 174.85(overlap 3) , 174.51 , 174,46 , 54.79 (overlap 3) , 45.77 (overlap 2) , 40.29 (overlap 3), 32.39 , 32.27 , 31.49 , 31,44 , 30.82 (d, J=41,1 Hz) , 30.56 , 29.97 , 28.94, 28.87 , 27.98 (d, J = 3.7 Hz), 27.54 (d, J=11,9 Hz) , 27.35 , 26.57 , 24.92 , 23.51 , 23,47 , 16.30 , 15.96 .

^{13}C NMR (126 MHz, Methanol-d_4) δ 173.43, 173.08 , 173.04 , 172.05 , 53.35 , 52.97 , 38.88 , 30.90 (d, *J* = 13.6 Hz), 30.11 , 29.4 (d, *J* = 41.1 Hz)29.20 , 29.12 , 29.10, 28.59 , 28.53 , 27.56 , 27.51 , 26.87 , 26.59 (d, *J* = 3.8 Hz), 26.13 (d, *J* = 11.7 Hz), 25.95 , 25.17 , 23.53 , 22.10 , 22.06 , 14.74 (d, *J* = 43.5 Hz).

MS: Calculated: 699,55680 Found: 699,55727 (M/2)

IR: 3423, 3250, 2931, 2854, 1448, 1122, 1008, 722

<u>Compound 6</u>

Triphenyl(3,14,25-trihydroxy-2,10,13,21,24-pentaoxo-31-(12-(triphenylphosphonio)dodecyl)-3,9,14,20,25,31-hexaazatritetracontan-31-ium-43-yl)phosphonium trichloride

Triphenylphosphoniumdodecyl bromide (1,5g mg; 2,5 mmol; 10 eq.), deferoxamine mesylate salt (170 mg; 0,25 mmol; 1eq.) and $NaHCO_3$ (1 g; 0,012 mol; 47 eq.) were dissolved in dry DMF (20 ml) and heated to 60°C and stirred at this temperature for 4h. After that reaction was cooled to rt and stirred overnight. Reaction process was monitored by TLC ($CHCl_3$/MeOH/NH_3; 80/20/2). Reaction was diluted by 20 ml of DCM, $NaHCO_3$ was filtrated of and solvents were evaporated. Product was dissolved in dichloromethane (5 mL) and precipitated by addition in ice cooled Et_2O (40 ml) and PE (40 ml) and decanted. Precipitate was dissolved in methanol/H_2O (5 ml) and filtrated through DOWEX (45 ml) and solvents were evaporated. Product was purified by column chromatography on silica gel ($CHCl_3$/MeOH/NH_3 100/15/1,5). Reaction afforded yellow oil of the formula 8 (47 mg, 63 %).

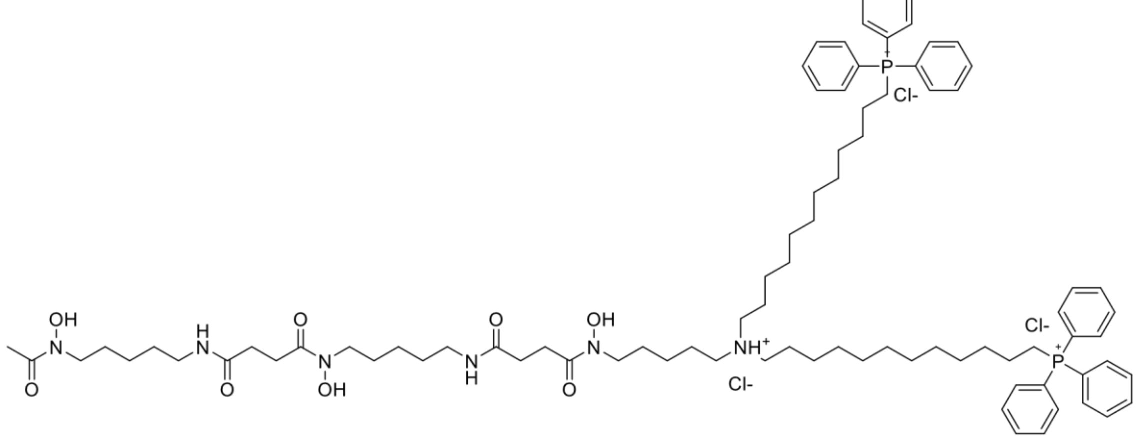

)

R_f 0.06 ($CHCl_3$/CH_3OH/NH_3 100:10:2)

^{1}H NMR (500 MHz, Methanol-d4) δ 8.02 – 7.66 (m, 30H), 3.61 (t, J = 6.9 Hz, 6H), 3.47 – 3.37 (m, 4H), 3.18 (m, 6H), 2.78 (t, J = 7.36 Hz, 4H), 2.55 (s, 4H), 2-47 (dt, J = 6.5, 2.2 Hz, 4H), 2.11 (s, 3H), 1.75 – 1.60 (m, 10H), 1.54 (m, 12H), 1.40 – 1.18 (m, 36H).

^{13}C NMR (126 MHz, Methanol-d_4) δ 173.47, 172.99 , 172.94 , 172.00 , 134.9 (d, J = 2.98 Hz), 133.4 (d, J = 9.03 Hz), 130.1 (d, J = 12.5 Hz), 118.65(d, J = 86.1Hz) , 53.60 , 53.30 , 38.89 , 30.17 , 29.31 , 29.28 , 29.23 , 29.19 , 29.04 , 28.58 , 28.54 , 27.55 , 27.22 , 26.13 , 25.95 , 25.62 , 25.12 , 24.16 , 23.51 (d, *J* = 2.9 Hz), 22.17 (d, *J* = 4.0 Hz), 21.48 , 21.07
MS: Calculated:1418,8945; Found: 709,44728 (m/z = 2)
IR: 2927, 2854, 1636, 1588, 1485, 1439, 1114, 996, 724, 691

<u>Compound 7</u>

2,2-dibenzyl-19,30,41-trihydroxy-20,23,31,34,42-pentaoxo-1-phenyl-13-(10-(tribenzylphosphonio)decyl)-13,19,24,30,35,41-hexaaza-2-phosphatritetracontane-2,13-diium trichloride

Tribenzyllphosphoniumdecyl bromide (1,57g mg; 2,6 mmol; 10 eq.), deferoxamine mesylate salt (170 mg; 0,26 mmol; 1eq.) and $NaHCO_3$ (1,03 g; 0,012 mol; 47 eq.) were dissolved in dry DMF (20 ml) and heated to 60°C and stirred at this temperature for 4h. After that reaction was cooled to rt and stirred overnight. Reaction process was monitored by TLC ($CHCl_3$/MeOH/NH_3; 80/20/2).
Reaction was diluted by 20 ml of DCM, $NaHCO_3$ was filtered off and solvents were evaporated. Crude product was dissolved. Solvents were in dichloromethane (5 mL) and precipitated by addition in ice cooled Et_2O (40 ml) and PE (40 ml) and decanted off and precipitate was dissolved in methanol/H_2O (5 ml) and slowly filtered through DOWEX (25 ml). Solvents were evaporated and product was purified by column chromatography on silica gel ($CHCl_3$/MeOH/NH_3 100/15/1),5. Reaction afforded yellow foam of the formula 9 (52 mg, 71 %).

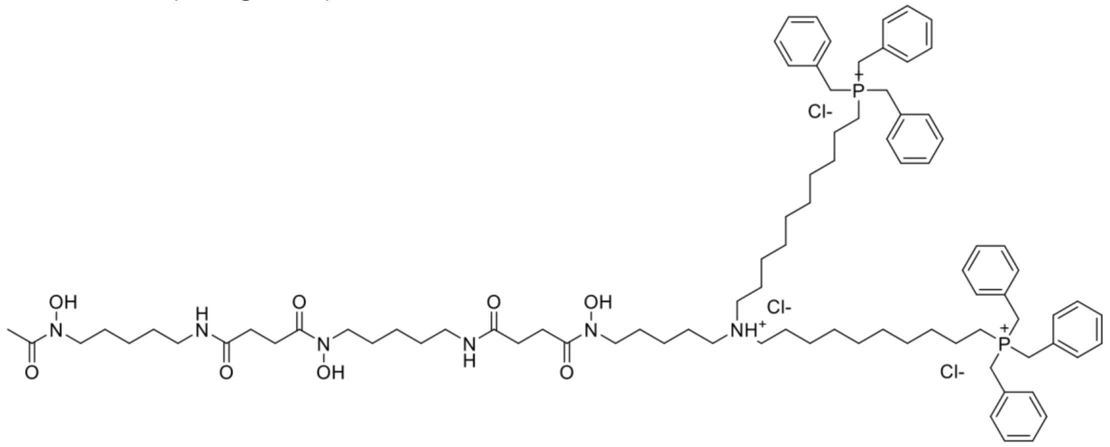

R_f 0.08 ($CHCl_3$/CH_3OH/NH_3 100:10:2)
^1H NMR (500 MHz, Methanol-d4) δ 7.52 – 7.21 (m, 30H), 3.80 (dd, J = 14.4, 6.7 Hz, 12H), 3.70 – 3.57 (m, 6H), 3.26 – 3.11 (m, 4H), 2.78 (t, J = 7.2 Hz, 4H), 2.64 (s, 6H), 2.48 (dd, J = 7.1, 3.4 Hz, 4H), 2.11 (s, 3H), 2.04 (d, J = 4.0 Hz, 4H), 1.74 – 1.60 (m, 6H), 1.55 (d, J = 8.2 Hz, 8H), 1.49 – 1.16 (m, 26H).
^{13}C NMR (126 MHz, Methanol-d4) δ 173,46 (overlap 2) , 173,45 (overlap) , 173,05 , 130,03 (d, J= 5,01 Hz) , 129,35 (d, J=2,81 Hz) , 128,42 (d, J= 3,18 Hz) , 127,78 (d, J= 7,95 Hz) , 53,57 , 53,21 , 38,89 , 30,35 (d, J=15,2 Hz) , 30,10 , 29,16 , 28,86 , 28,58 , 28,55 , 28,38 , 27,54 , 27,52 , 27,11 , 26,06 , 25,94 , 25,41 , 24,00 , 23,52 , 23,50 , 20,90 (d, J= 5,1 Hz) , 18,47 (d, J= 45,9 Hz).
MS: Calculated: 1446,9258; Found:723,46320 (M/Z=2)
IR: 3063, 1636, 1551, 1496, 1455, 1257, 1075, 702

<u>Compound 8</u>

3,14,25-trihydroxy-42,42-dioctyl-2,10,13,21,24-pentaoxo-31-(10-(trioctylphosphonio)decyl)-3,9,14,20,25,31-hexaaza-42-phosphapentacontane-31,42-diium trichloride

Trioctylbromodecylfosfonium bromide (1,73g mg; 2,6 mmol; 10 eq.), deferoxamine mesylate salt (170 mg; 0,26 mmol; 1eq.) and $NaHCO_3$ (1,03 g; 0,012 mol; 47 eq.) were dissolved in dry DMF (20 ml) and heated to 60°C while stirred for 2,5h. After that reaction was cooled to rt and stirred overnight. Reaction progress was monitored by TLC ($CHCl_3$/MeOH/NH_3; 80/20/2). Reaction was diluted by 20 ml of DCM, $NaHCO_3$ was filtered off and solvents were evaporated. Crude product was dissolved in methanol/H_2O (5 ml) and filtered through DOWEX (25 ml) and solvents were evaporated. Product as chloride was

purified by column chromatography on silica gel ($CHCl_3$ $CHCl_3$/MeOH/NH_3 100/15/1,5). Reaction afforded yellow foam of bisphosphonium of the formula 10 (34 mg, 51 %) and 7 mg (5%) of monophosphonium.

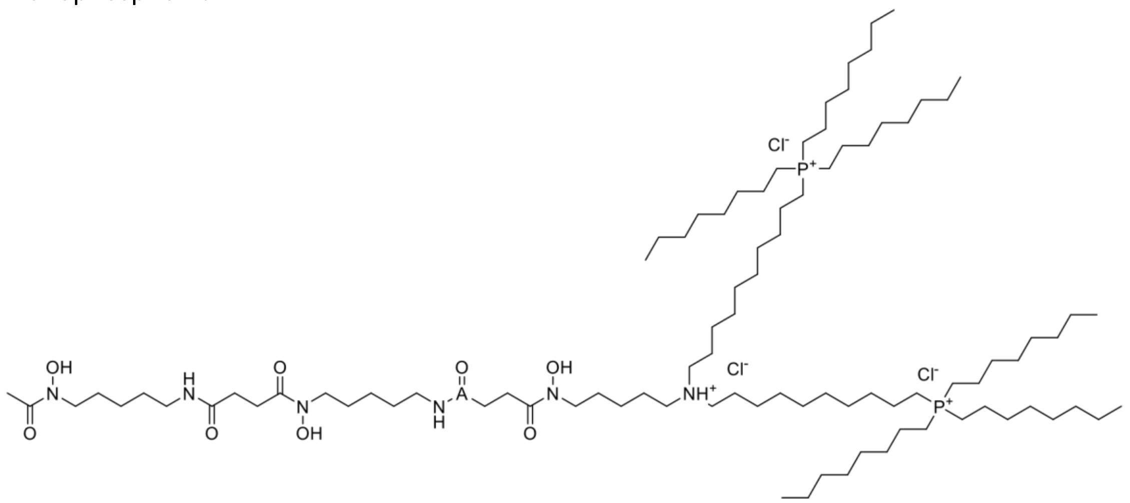

R_f 0.15 ($CHCl_3$/CH_3OH/NH_3 100:10:2)
1H NMR (500 MHz, Methanol-d4) δ 3.67 – 3.58 (m, 6H), 3.56 (dt, J = 7.8, 6.5 Hz, 4H), 3.21 – 3.15 (m, 6H), 2.78 (t, J = 7.2 Hz, 4H), 2.48 (dd, J = 7.5, 4.9 Hz, 4H), 2.22 (ddd, J = 16.7, 10.4, 6.7 Hz, 16H), 2.11 (s, 3H), 1.83 – 1.70 (m, 10H), 1.63 – 1.32 (m, 120H), 0.92 (t, J = 6.6 Hz, 18H).
13C NMR (126 MHz, Methanol-d4) δ 173,47 , 173,44 , 173,06 , 173,03 , 173,01 , 53.38,53.26,53.22, 44,35(2) , 38,87(3) , 32,24 , 32,26 , 31,52 , 30,58 , 30,37 (J=48,0 Hz) , 30,06 , 29,4-28,9 , 28,72 , 28,56 , 28,49 , 27,51, 26,46 , 26,06 , 25,93 , 25,54 , 23,49 (J= 1,8 Hz) , 22,29 , 22,19 , 22,10 , 21,07 , 21,04 , 20,94 , 20,91 , 17,83 (J= 48,0 Hz) , 13,02.
MS: Calculated: 790.20129; Found:790.20123 (M/Z=2)
IR:3069, 2927, 1641, 1544, 1461, 723

<u>Compound 9</u>

13-(10-(dimethyl(phenyl)phosphonio)decyl)-19,30,41-trihydroxy-2-methyl-20,23,31,34,42pentaoxo-2-phenyl-13,19,24,30,35,41-hexaaza-2-phosphatritetracontan-2-ium trichloride

Dimethylpheny,bromodecyllfosfonium bromide (1,3g mg; 2,9 mmol; 10 eq.), deferoxamine mesylate salt (190 mg; 0,30 mmol; 1eq.) and $NaHCO_3$ (1,17 g; 0,014 mol; 47 eq.) were dissolved in dry DMF (20 ml) and heated to 60°C while stirred 4h. After that reaction was cooled to rt and stirred overnight. Reaction progress was monitored by TLC ($CHCl_3$/MeOH/NH_3; 80/20/2). Reaction was diluted by 20 ml of DCM, $NaHCO_3$ was filtered off and solvents were evaporated. Crude product was dissolved in methanol/H_2O (5 ml) and filtered through DOWEX (25 ml). All solvents were evaporated and product as chloride was purified by column chromatography on silica gel ($CHCl_3$ -$CHCl_3$/MeOH/NH_3 100/15/1,5). Reaction afforded yellow foam of the structure 12 (54 mg, 57 %).

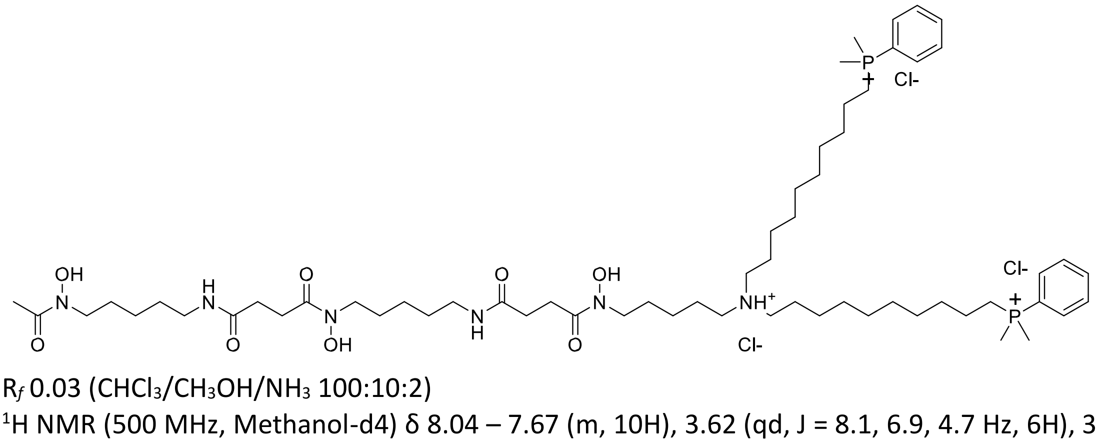

R_f 0.03 ($CHCl_3$/CH_3OH/NH_3 100:10:2)
^{1}H NMR (500 MHz, Methanol-d4) δ 8.04 – 7.67 (m, 10H), 3.62 (qd, J = 8.1, 6.9, 4.7 Hz, 6H), 3.23 – 3.15 (m, 4H), 3.04 (s, 6H), 2.78 (s, 4H), 2.57 – 2.44 (m, 12H), 2.24 (dd, J = 12.7, 3.1 Hz, 12H), 2.11 (s, 3H), 1.75 – 1.27 (m, 40H).
^{13}C NMR (126 MHz, Methanol-d4) δ 173,44 , 173,41 , 173,18 , 173,04 , 134,15 (d, J=2,73) , 130,97

(d, J= 10,32 Hz) , 129,70 (d, J= 12,52 Hz) , 120,49 (d, J= 85,18 Hz) , 53,02 , 52,65 , 38,87 , 32,18 , 30,21 , 30,08 , 29,94 , 29,34 , 29,09 , 28,95 , 28,90 , 28,56 , 27,51 , 27,39 , 26,43 , 25,92 , 23,49 , 23,43 , 23,39 , 23,02 , 21,25 , 21,21 , 21,17 , 5,73 (d, J= 54,73 Hz).
MS:Calculated: 557,38465; Found: 557,38488 (M/Z=3)
IR: 3434(m), 3260(m), 3063(w(), 2928(s), 2856(m), 1638(s), 1548(m), 1457(m), 1438(m), 1122(m), 998(m), 749(m), 692(m).

Compound 10

triphenyl(10-(5-(8,19,30-trihydroxy-9,12,20,23,31-pentaoxo-2-((1-(10-(triphenyl phosphonio)decyl)-1H-1,2,3-triazol-5-yl)methyl)-2,8,13,19,24,30-hexaazadotriacontyl)-1H-1,2,3triazol-1-yl)decyl)phosphonium

*Bispropargyldeferoxamine **I** (60mg, 0.094 mmol) together with sodium ascorbate (4 mg, 0.020 mmol) and CuSO4.5H2O (10 mg, 0.040 mmol) was placed in flask and **(10-azidodecyl)triphenylphosphonium bromide **II** (100 mg, 0.190 mmol) dissolved in DMF (1mL) and water (1mL) was added. Reaction mixture was stirred under 60°C for 1 hour. Reaction was monitored with TLC (PMA stain, chloroform/ methanol/ ammonium 80/20/2, Rf 0.1). When the reaction was completed, solvents were evaporated, crude material dried and purified with column of silicagel (eluent: chloroform/ methanol/ammonium 80/20/2) to get product in the form of light brown oil 138 mg (87%) of the formula 20.

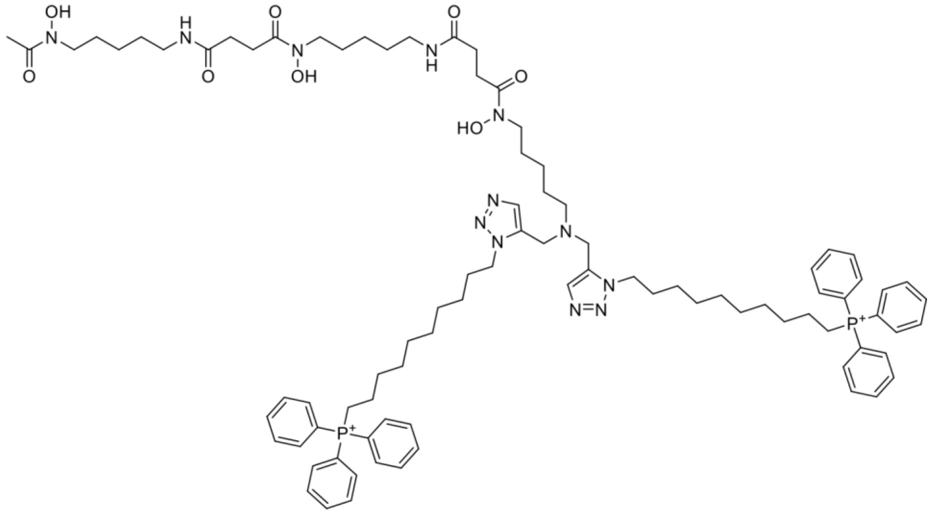

R_f 0.04 ($CHCl_3$/CH_3OH/NH_3 100:10:2)
1H NMR (500 MHz, Methanol-d4) δ 8.05 (s, 2H), 7.94 – 7.75 (m, 30H), 4.43 (t, J = 6.9 Hz, 4H), 3.87 (s, 4H), 3.66 – 3.54 (m, 6H), 3.49 – 3.40 (m, 4H), 3.24 – 3.13 (m, 4H), 2.83 – 2.74 (m, 4H), 2.52 (m, 2H), 2.50 – 2.44 (m, 4H), 2.12 (s, 3H), 1.92 (t, J = 7.2 Hz, 4H), 1.73 – 1.60 (m, 10H), 1.60 – 1.48 (m, 10H), 1.41 – 1.21 (m, 20H).
13C NMR (126 MHz, Methanol-d4) δ 173.44, 173.40, 173.04, 173.00, 172.06, 134.86 (d, J = 2.8 Hz), 133.42 (d, J = 9.9 Hz), 130.12 (d, J = 12.6 Hz), 124.35, 118.62 (d, J = 86.2 Hz), 52.6, 52.4, 49.95 38.87, 30.23, 30.16, 30.10, 29.84, 28.80 (d, J = 10.47 Hz), 28.58, 28.52, 28.40 (d, J = 14.65 Hz), 27.60, 27.55, 26.00, 25.95, 23.84, 23.52, 22.17, 22.13, 21.3 (d, J = 50.70 Hz), 18.88.
HRMS calcd for C87H122O8N12P2 (m/z=2) 762.44864 found: 762.44881
IR (KBr pellet): ν = 3421, 3259, 2927, 2855, 2212, 1636, 1586, 1546, 1483, 1458, 1438, 1416, 1319, 1257, 1213, 1193, 1160, 1113, 1052, 1025, 996, 792, 750, 723, 690, 531, 509, 414. **N*1-(5-(di(prop-2-yn-1-yl)amino)pentyl)-N1-hydroxy-N4-(5-(N-hydroxy-4-((5-(N-hydroxy acetamido)pentyl)amino)-4-oxobutanamido)pentyl)succinimide (**I**)

Deferoxamine mesylate (100 mg, 0.1523 mmol) was dissolved in DMF (2 mL) and NaHCO3 (384 mg, 4.571 mmol) was added in one portion followed with propargyl bromide (33 µl, 0.306 mmol) as 80% solution in toluene. Reaction mixture was stirred under 80°C for 4 hours and monitored with TLC (PMA stain, chloroform/ methanol/ammonium 80/20/2, Rf 0.45). When reaction was completed reaction mixture was allowed to cool to laboratory temperature, dichloromethane (5 ml) was added and reaction mixture was filtered, evaporated and dried. Crude product was purified with column of silicagel (eluent: chloroform/ methanol/ammonium

80/20/2) to get product in the form of white/yellow solid 69 mg (71%)

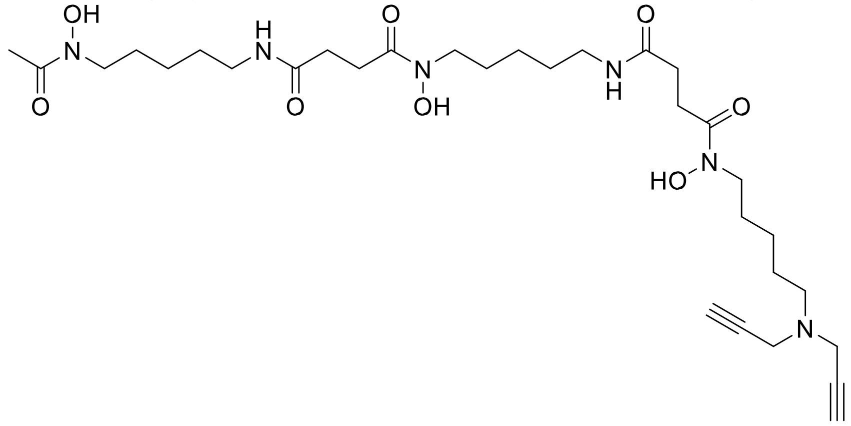

^{1}H NMR (500 MHz, Methanol-d4) δ 4.58 (s, 2H), 3.60 (t, J = 7.0 Hz, 6H), 3.44 (s, 3H), 3.43 (s, 2H), 3.17 (t, J = 7.0 Hz, 4H), 2.77 (t, J = 7.2 Hz, 4H), 2.63 (t, J = 2.4 Hz, 2H), 2.58 – 2.52 (m, 2H), 2.49 – 2.42 (m, 4H), 2.09 (s, 3H), 1.71 – 1.59 (m, 6H), 1.58 – 1.47 (m, 7H), 1.40 – 1.27 (m, 8H).
^{13}C NMR (126 MHz, Methanol-d4) δ 174.92, 174.47, 174.42, 173.51, 79.03, 74.84, 53.76, 42.69, 40.28, 31.53, 31.48, 29.97, 29.93, 28.95, 28.92, 27.68, 27.49, 27.33, 25.39, 24.90, 24.87, 20.24. HRMS calcd for C31H53O8N6 637.39194 found: 637.39240, calcd for C31H53O8N6Na 659.37388 found: 659.37393.
IR (KBr pellet): ν = 3323, 3290, 3143, 2929, 2857, 2113, 1654, 1623, 1565, 1457, 1268, 1253, 1192, 1159, 960, 726, 676.

(10-azidodecyl)triphenylphosphonium bromide (II**)

(10-bromodecyl)triphenylphosphonium bromide (500 mg, 0.889 mmol) was dissolved in DMF (15 mL) and sodium azide (575 mg, 8.845 mmol) was added in one portion under stirring. Mixture was heated under 80°C overnight. Reaction was monitored with TLC (PMA stain, chloroform/ methanol/ammonium 80/20/2, Rf of starting material is the same as of the product 0-0.2, but slightly different color occurs during heating the plate). Sodium azide was filtered off after cooling to laboratory temperature, DMF was evaporated and crude material was purified with short column of silicagel (eluent: chloroform/methanol 10/1). Product was obtained in the form of yellowish oil 450 mg (96 %)

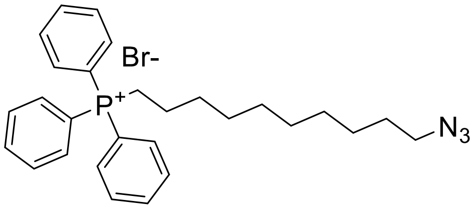

^{1}H NMR (500 MHz, Methanol-d4) δ 7.98 – 7.76 (m, 15H), 3.46 (ddd, J = 16.4, 8.2, 5.4 Hz, 2H), 3.30 (t, J = 6.8 Hz, 2H), 1.71 (m, 2H), 1.60 (m, 4H), 1.46 – 1.22 (m, 10H).
^{13}C NMR (126 MHz, Methanol-d4) δ 134.86 (d, J = 2.85 Hz), 133.43 (d, J = 9.9 Hz), 130.13 (d, J = 12.6 Hz), 118.62 (d, J = 86.0 Hz), 51.04, 30.17 (d, J = 16.2 Hz), 28.98, 28.84, 28.76, 28.46 (d, J = 4.9 Hz), 26.36, 22.14 (d, J = 4.6 Hz), 21.51, 21.11.
HRMS calcd for C28H35N3P 444.25631 found: 444.25643.
IR (KBr pellet): ν = 3395, 3052, 2926, 2854, 2094, 2003, 1587, 1485, 1438, 1345, 1256, 1190, 1162, 1113, 996, 790, 751, 723, 691, 616, 533, 509.

<u>Compound 11</u>

(10-(3,5-bis(2-hydroxyphenyl)-1H-1,2,4-triazol-1-yl)decyl)triphenylphosphonium bromide

(10-bromodecyl)triphenylphosphonium bromide (750 mg, 1.334 mmol) was heated with hydrazine (6 ml) for 2 hours. Reaction mixture was cooled to laboratory temperature, diluted with H_2O (50 ml) and

extracted 2 x with DCM and dried over $MgSO_4$. Crude material was concentrated, dried under vacuum, diluted with ethanol (6 ml) and 2-(2-hydroxyphenyl)-4H-benzo[e][1,3]oxazin4-one (377 mg, 1.576 mmol) was added and reaction mixture was refluxed for 1.5 hours. Reaction mixture was concentrated under vacuum and purified by column chromatography (toluene: methanol, gradient 100:5 – 100:15) to yield product (330 mg, 30 %) of as light yellow foam.

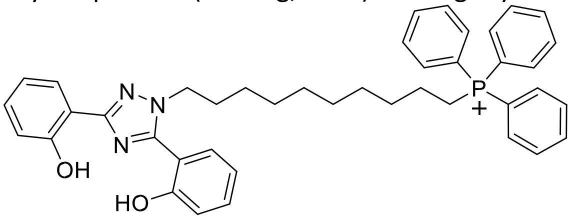

R_f 0.20 ($CHCl_3$/CH_3OH/NH_3 100:10:2)
^{1}H NMR (600 MHz, Chloroform-*d*) δ 10.55 (s, 1H), 8.06 (dd, *J* = 7.8, 1.7 Hz, 1H), 7.83 – 7.70 (m, 9H), 7.65 (td, *J* = 8.0, 3.4 Hz, 6H), 7.48 – 7.43 (m, 2H), 7.35 (ddd, *J* = 8.5, 7.2, 1.6 Hz, 1H), 7.31 – 7.25 (m, 2H), 7.22 – 7.16 (m, 1H), 6.99 (ddd, *J* = 7.3, 6.5, 1.0 Hz, 2H), 6.95 (td, *J* = 7.5, 1.1 Hz, 1H), 4.28 (t, *J* = 7.4 Hz, 2H), 3.64 (td, *J* = 12.6, 5.1 Hz, 2H), 1.96 (p, *J* = 7.5 Hz, 2H), 1.61 – 1.50 (m, 4H), 1.35 – 1.28 (m, 2H), 1.28 – 1.10 (m, 8H).
^{13}C NMR (151 MHz, Chloroform-*d*) δ 159.10, 156.71, 156.55, 152.15, 134.96 (d, *J* = 3.0 Hz), 133.58 (d, *J* = 10.7 Hz), 132.20, 130.85, 130.43 (d, *J* = 12.5 Hz), 128.23, 126.93, 119.48, 119.40, 118.27 (d, *J* = 85.8 Hz), 117.97, 116.90, 114.28, 112.61, 50.23, 30.12 (d, *J* = 15.8 Hz), 29.28, 28.73 (overlap), 28.68 (d, *J* = 1.1 Hz), 26.18, 22.80, 22.51, 22.48 (overlap), 22.47. HRMS calculated for $C42H45O2N3P^+$: 654.32439, found: 654.32443.

Compound 12

(8-(4-(3,5-bis(2-hydroxyphenyl)-1H-1,2,4-triazol-1-yl)benzamido)octcyl) triphenylphosphonium chloride

Deferasirox (11.4 mg, 0.031 mmol) with 8-aminooctylphosphonium chloride hydrochloride (15 mg, 0.033 mmol), N-(3-dimethylaminopropyl)-N′-ethylcarbodiimide hydrochloride (EDC) (88 mg, 0.46 mmol) and N,N-diisopropylethylamine (DIPEA) (0.32 ml, 1.86 mmol) were dissolved in dimethylformamide (DMF) (3 ml) and reaction was stirred for 72 hours. Solvents were evaporated, mixture was dissolved in methanol (MeOH) and extracted with citric acid (5 %), brine and dichloromethane (DCM). Pure product (2.5 mg) was isolated by preparative HPLC chromatography (C18 reverse phase) (5% MeOH/H_2O) as light oil.
^{1}H NMR (500 MHz, Chloroform-d) δ 10.92 (bs, OH, 1H), 10.21 (bs, OH, 1H), 8.44 (bs, NH, 1H), 8.12 (t, J = 7.9 Hz, 2H), 7.79 – 7.68 (m, 6H), 7.60 (s, 7H), 7.44 (d, J = 6.9 Hz, 2H), 7.28 (t, J = 7.9 Hz, 1H), 7.25 (dd, J = 26.3, 7.7 Hz, 2H), 7.10 (d, J = 7.7 Hz, 1H), 7.03 (m, 2H), 6.74 (t, J = 7.4 Hz, 1H), 3.45 (m, 4H), 1.79 – 1.42 (m, 6H), 1.24 (m, 6H).
^{13}C NMR (126 MHz, Chloroform-d) δ 166.41, 159.66, 157.12, 156.66, 152.15, 139.80, 135.72, 135.30, 133.42 (d, J = 9.9 Hz), 132.57, 131.11, 130.77 (d, J = 12.4 Hz), 129.18, 128.02, 127.12, 124.99, 119.73, 118.00 (d, J = 85.74 Hz), 117.09, 117.05, 113.35, 111.33, 40.64, 30.19, 30.05, 28.44, 28.50, 28.41, 26.17, 22.83, 22.51, 22.40.
HRMS calculated for $C45H46O3N4P^+$: 745.36150, found: 745.36023.

Compound 13

(6-(4-(3,5-bis(2-hydroxyphenyl)-1H-1,2,4-triazol-1-yl)benzamido)hexyyl) triphenylphosphonium chloride

Deferasirox (11.4 mg, 0.31 mmol) with 6-aminodecylphosphonium chloride hydrochloride (15.0 mg, 0.035 mmol), N-(3-dimethylaminopropyl)-N′-ethylcarbodiimide hydrochloride (EDC) (88 mg, 0.46 mmol) and N,N-diisopropylethylamine (DIPEA) (0.32 ml, 1.86 mmol) were dissolved in dimethylformamide (DMF) (3 ml) and reaction was stirred for 72 hours. Solvents were evaporated, mixture was dissolved in methanol (MeOH) and extracted with citric acid (5 %), brine and dichloromethane (DCM). Pure product (2 mg) was isolated by preparative HPLC chromatography

(C18 reverse phase) (5% MeOH/H_2O) as light oil

^{1}H NMR (500 MHz, Chloroform-d) δ 10.87 (bs, OH, 1H), 10.17 (bs, OH, 1H), 8.40 (bs, NH, 1H), 8.11 (t, J = 7.9 Hz, 2H), 7. – 7.58 (m, 6H), 7.62 (s, 7H), 7.40 (d, J = 6.9 Hz, 2H), 7.38 (t, J = 7.9 Hz, 1H), 7.22 (dd, J = 26.3, 7.7 Hz, 2H), 7.11 (d, J = 7.7 Hz, 1H), 7.02 (m, 2H), 6.73 (t, J = 7.4 Hz, 1H), 3.46 (m, 4H), 1.73 – 1.40 (m, 4H), 1.24 (m, 4H).

^{13}C NMR (126 MHz, Chloroform-d) δ 166.58, 159.42, 157.10, 156.22, 152.14, 139.67, 135.92, 135.30, 133.40 (d, J = 9.9 Hz), 132.53, 131.10, 130.65 (d, J = 12.4 Hz), 129.10, 128.00, 127.10, 124.85, 119.49, 118.00 (d, J = 85.74 Hz), 117.15, 117.05, 113.19, 111.33, 4058, 30.19, 30.39, 28.44, 28.74, 28.31, 22.51, 22.40.

HRMS calculated for C43H42O3N4P$^+$: 717.36150, found: 717.36009.

Compound 14

(10-(4-(3,5-bis(2-hydroxyphenyl)-1H-1,2,4-triazol-1-yl)benzamido)decyl) triphenylphosphonium chloride

Deferasirox (114 mg, 0.31 mmol) with 10-aminodecylphosphonium chloride hydrochloride (150 mg, 0.31 mmol), N-(3-dimethylaminopropyl)-N′-ethylcarbodiimide hydrochloride (EDC) (88 mg, 0.46 mmol) and N,N-diisopropylethylamine (DIPEA) (0.32 ml, 1.86 mmol) were dissolved in dimethylformamide (DMF) (3 ml) and reaction was stirred for 72 hours. Solvents were evaporated, mixture was dissolved in methanol (MeOH) and extracted with citric acid (5 %), brine and dichloromethane (DCM). Pure product (25 mg, 10 %), isolated as two rotamers was obtained by column chromatography (2% MeOH/$CHCl_3$) as light yellow foam.

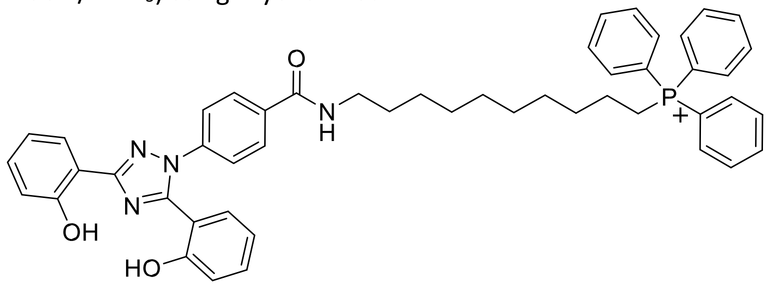

R_f 0.20 ($CHCl_3$/CH_3OH/NH_3 100:10:2)

^{1}H NMR (500 MHz, Chloroform-d) δ 10.96 (bs, OH, 1H), 10.16 (bs, OH, 1H), 8.40 (bs, NH, 1H), 8.12 (t, J = 7.9 Hz, 2H), 7.79 – 7.68 (m, 6H), 7.65 (s, 7H), 7.42 (d, J = 6.9 Hz, 2H), 7.33 (t, J = 7.9 Hz, 1H), 7.25 (dd, J = 26.3, 7.7 Hz, 2H), 7.11 (d, J = 7.7 Hz, 1H), 7.00 (m, 2H), 6.74 (t, J = 7.4 Hz, 1H), 3.46 (m, 4H), 1.79 – 1.48 (m, 6H), 1.26 (m, 10H).

^{13}C NMR (126 MHz, Chloroform-d) δ 166.48, 159.61, 157.18, 156.65, 152.14, 139.81, 135.84, 135.10, 133.52 (d, J = 9.9 Hz), 132.60, 131.39, 130.52 (d, J = 12.4 Hz), 129.14, 128.79, 127.31, 124.61, 119.65, 19.24, 118.15 (d, J = 85.74 Hz), 117.95, 117.05, 113.65, 111.91, 40.17, 30.19, 30.06, 29.69, 29.00, 28.59, 28.50, 28.46, 26.61, 22.83, 22.51, 22.47.

HRMS calculated for C49H50O3N4P$^+$: 773.36150, found: 773.36128.

Compound 15

(10-(2,4-bis(2-hydroxyphenyl)-1H-imidazol-1-yl)decyl)triphenylphosphonium chloride

Compound **III** (100 mg, 0.397 mmol), Compound **IV** (389 mg, 0.793 mmol), O-(benzotriazol-1-yl)N,N,N′,N′-tetramethyluronium tetrafluoroborate (TBTU) (255 mg, 0.794 mmol) and triethylamine (Et_3N) (0.55 ml, 3.9 mmol) were dissolved in DMF (1 ml) and reaction mixture was stirred for 2 h. The reaction mixture was added dropwise into ice cooled diethylether (Et_2O) (50 ml) and oily precipitate formed on the walls of flask upon stirring in ice-bath. Solvent was decanted off and precipitate was dissolved in methanol (3mL) and diluted with half saturated solution of NH_4Cl (10 mL). Resulting milky solution was extracted DCM (3 x 15 mL). Combined organic layer was dried over $MgSO_4$ and concentrated under vacuum. The identical extraction procedure (3 mL of methanol, 10 mL NH_4Cl, 3 x 15 mL DCM) was repeated two more times. The crude product was purified by column chromatography (chloroform/methanol gradient 0-10% of methanol) to afford product (170 mg, 62 %) as a yellowish foam.

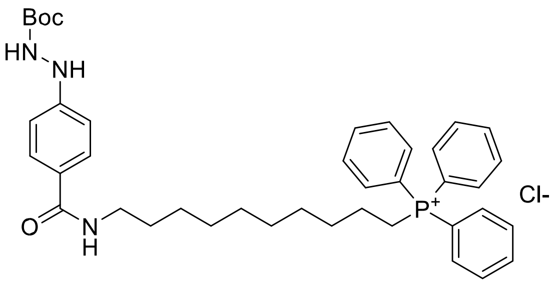

R_f 0.80 ($CHCl_3/CH_3OH/NH_3$ 100:10:2)
^{1}H NMR (600 MHz, Methanol-d4) δ 7.93 – 7.74 (m, 15H), 7.69 (d, J = 8.7 Hz, 2H), 6.81 – 6.75 (m, 2H), 3.44 – 3.37 (m, 2H), 1.67 (h, J = 8.2 Hz, 2H), 1.59 (q, J = 6.9 Hz, 2H), 1.55 (t, J = 7.5 Hz, 2H), 1.51 (s, 6H), 1.33 (d, J = 24.7 Hz, 12H).
^{13}C NMR (151 MHz, Methanol-d4) δ 168.69, 157.33, 152.31, 134.85, 133.39 (d, J = 10.0 Hz), 130.09 (d, J = 12.6 Hz), 128.20, 124.42, 118.88, 118.31, 110.92, 39.39, 30.11 (d, J = 16.1 Hz), 29.14, 28.93, 28.81 (d, J = 7.1 Hz), 28.34, 27.23, 26.54, 22.13 – 21.97 (m), 21.32, 20.99. HRMS calculated for C40H51O3N3P$^+$: 652.36626, found: 652.36596.

4-(2-(tert-butoxycarbonyl)hydrazineyl)benzoic acid (Compound **III**)

4-hydrazinobenzoic acid (500 mg, 3.286 mmol) and Boc anhydride (720 mg, 3.299 mmol, Boc = di(tert-butyl)dicarbonate) were dissolved in DMF (4 ml), DIPEA (1 ml) was added and reaction mixture was stirred for 1 hour. Reaction mixture was concentrated and dried under vacuum. Crude material was purified on column chromatography (chloroform: methanol, 100:10) to get product (690 mg, 79 %) in the form of yellowish foam.

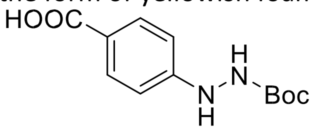

^{1}H NMR (600 MHz, Methanol-d4) δ 7.88 – 7.76 (m, 2H), 6.78 – 6.65 (m, 2H), 1.46 (s, 6H).
^{13}C NMR (151 MHz, Methanol-d4) δ 170.37, 158.67, 154.93, 132.45, 121.78, 112.01, 81.54, 28.63. HRMS calculated for C12H17O4N2$^+$: 253.11828, found: 253.11779.

(10-ammoniodecyl)triphenylphosphonium chloride hydrochloride (Compound **IV**)

(10-bromodecyl)triphenylphosphonium bromid (9.26 g, 16.448 mmol) was dissolved in 7M solution of ammonia in methanol (60 ml) and the reaction mixture was stirred at 50 ºC. After 6 h, additional ammonia in methanol (40 ml) was added and the reaction mixture was heated for additional 24 h at 50 ºC. The reaction mixture was concentrated under reduced pressure, and crude product was purified using column chromatography on silicagel (200 ml) (chloroform/methanol gradient 0-10% of methanol). The product was acidified with HCl (36%, 5 ml) and filtered through Dowex 1x8 in chloride form (50 g) to obtain desired (10aminodecyl)triphenylphosphonium chloride hydrochloride (5.135 g, 63%).

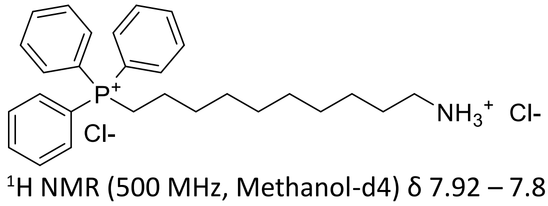

^{1}H NMR (500 MHz, Methanol-d4) δ 7.92 – 7.85 (m, 3H), 7.85 – 7.68 (m, 12H), 3.46 – 3.37 (m, 2H), 2.90 (t, J = 7.5 Hz, 2H), 1.72 – 1.60 (m, 4H), 1.56 (p, J = 7.5 Hz, 2H), 1.43 –1.20 (m, 12H)
^{13}C NMR (126 MHz, Methanol-d4) δ 136.23 (d, J = 3.0 Hz), 134.79 (d, J = 9.9 Hz), 131.51 (d, J = 12.5 Hz), 120.00 (d, J = 86.3 Hz), 40.76, 31.59 (d, J = 16.2 5 Hz), 30.29 (2C), 30.12, 29.89, 28.53, 27.42, 23.57 (d, J = 4.4 Hz), 22.68 (d, J = 51.1 Hz).
IR (KBr pellet): ν = 3051, 3007, 2927, 2854, 2006, 1825, 1601, 1587, 1485, 1465, 1438, 1402, 1337, 1318, 1189, 1161, 1113, 996, 751, 723, 691.
HRMS calculated for C28H37NP$^+$: 418.26581, found: 418.26567.
